# Metabolite–neuro–immune relay in chronic pain: spatial–temporal lactate, succinate and itaconate signalling as drivers of glial reprogramming and neuronal sensitisation

**DOI:** 10.3389/fphar.2026.1786751

**Published:** 2026-04-28

**Authors:** Zhen Hu, Quan Ji, Qianqi Xiong, Youzhi Ning, Yong Rao, Jia Fu, Lu Wang, Xia Xiong, Cehua Ou, Yue Zhang

**Affiliations:** 1 Department of Pain Management, The Affiliated Hospital, Southwest Medical University, Luzhou, China; 2 Department of Anesthesiology Management, Sichuan Second Hospital of T.C.M, Chengdu, China; 3 Department of Anesthesiology, The Affiliated Hospital, Southwest Medical University, Luzhou, China; 4 Department of Dermatology, The Affiliated Hospital, Southwest Medical University, Luzhou, China

**Keywords:** chronic pain, signaling metabolites, microglia, biomarkers, patient stratification

## Abstract

Chronic pain is sustained by coupled neuronal hyperexcitability and neuroinflammation, yet prevailing frameworks incompletely explain why similar injuries diverge toward recovery or persistent sensitisation. Growing evidence indicates that lactate, succinate and itaconate act as signalling metabolites that shape glial state transitions and nociceptive circuit gain. Here, we synthesise preclinical and emerging clinical findings and propose a metabolite–neuro–immune relay model in which metabolic perturbations in astrocytes, microglia and peripheral immune cells generate characteristic lactate–succinate–itaconate patterns; glia decode these cues into pro-inflammatory or pro-resolving programs; and the resulting cytokines and physicochemical changes remodel dorsal root ganglion and spinal dorsal horn circuits. We highlight how spatially restricted metabolic microdomains and temporally phased shifts from transient bursts to stable immunometabolic reprogramming can sustain self-reinforcing neuroimmune loops. We then outline mechanism-guided therapeutic opportunities, including modulation of pathological glycolysis, lactate and acidosis-targeted microenvironment remodelling, succinate receptor 1 blockade and augmentation of the IRG1–itaconate–NRF2 axis using precision delivery approaches. This framework links molecular immunometabolism with circuit plasticity and offers testable targets for stage-aware analgesic development.

## 1 Introduction

Chronic pain is a pervasive and disabling condition, affecting a substantial proportion of the global population and undermining quality of life, work capacity and socioeconomic stability. Over the past decades, work in animal models and patients has established that neuronal hyperexcitability, maladaptive synaptic plasticity and neuroinflammation together underpin the transition from acute to chronic pain (Echeverria-Villalobos et al., 2023; Kiritoshi et al., 2024; Xiong et al., 2024). Classical paradigms, exemplified by tumour necrosis factor-alpha (TNF-α)–induced enhancement of sodium channel activity in sensory neurons, firmly linked inflammatory signals to nociceptor sensitisation (Tyagi et al., 2024; de Macedo et al., 2019). Yet these frameworks only partially explain why some individuals with apparently similar injuries develop persistent pain whereas others recover, and why a large fraction of patients remain refractory to conventional analgesics that target single transmitters or ion channels. These limitations have prompted a search for integrative models that place neuronal circuits within their broader immune and tissue environment.

Within this broader view, metabolism has emerged as a critical but previously underappreciated dimension. Metabolites were long regarded as passive intermediates or end products of bioenergetic pathways, useful mainly as biomarkers of physiological or pathological states. Recent work instead identifies a subset of signalling metabolites that actively shape intercellular communication, immune activation and neuronal plasticity. Among these, lactate, succinate and itaconate have attracted particular attention. Lactate, produced by glycolytic astrocytes and immune cells, can modulate microglial inflammatory programmes through hypoxia-inducible factor-1α (HIF-1α) and nuclear factor-κB (NF-κB) signalling and influence nociceptor excitability via acid-sensing ion channels (Zhang et al., 2025; Kong et al., 2022). Succinate, acting through the G protein-coupled succinate receptor 1 (SUCNR1) and via intracellular accumulation, stabilises HIF-1α, promotes nucleotide-binding oligomerisation domain, leucine-rich repeat and pyrin domain-containing protein 3 (NLRP3) inflammasome activation and enhances interleukin-1β (IL-1β) production (Swanson et al., 2019; Luo et al., 2024). By contrast, the immunometabolite itaconate, generated by immune-responsive gene 1 (IRG1), inhibits succinate dehydrogenase, activates nuclear factor erythroid 2-related factor 2 (NRF2) and suppresses NLRP3, thereby exerting predominantly anti-inflammatory and cytoprotective effects (Mills et al., 2018). Together, these findings argue that lactate, succinate and itaconate are not mere bystanders but active drivers or brakes in pain chronification.

**FIGURE 1:**
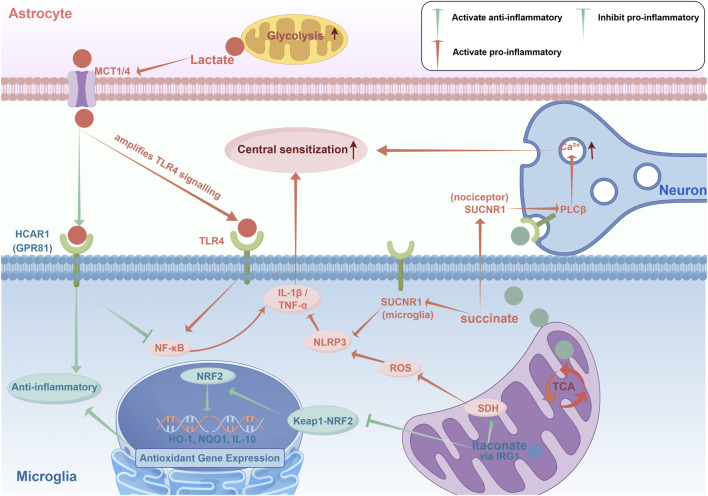
Metabolite–neuro–immune relay in chronic pain. Astrocyte-derived lactate and immune/glial-derived succinate can amplify microglial inflammatory signaling and nociceptor excitability, whereas IRG1-derived itaconate provides an intrinsic counter-regulatory brake. Lactate is exported through MCT1/4 and can potentiate TLR4–NF-κB signaling to promote IL-1β and TNF-α, reinforcing central sensitization, while HCAR1-mediated effects are likely context- and cell-type dependent. Succinate engages SUCNR1 on microglia and nociceptors:in microglia, this drives ROS–NLRP3 inflammasome activation to amplify pro-inflammatory cytokine production; in nociceptors, SUCNR1 coupling triggers phospholipase Cβ (PLCβ) activation, mobilizing intracellular Ca^2+^ and directly promoting neuronal hypersensitivity to consolidate central sensitization. Itaconate, generated via IRG1, inhibits succinate dehydrogenase (SDH) and activates KEAP1–NRF2-dependent antioxidant and pro-resolving gene expression (e.g., HO-1, NQO1, IL-10), counteracting pathological metabolic signaling Figure 2.

In this Review, we propose a metabolite–neuro–immune relay model of chronic pain. In this conceptual framework, upstream metabolic perturbations in astrocytes, microglia and peripheral immune cells generate characteristic patterns of lactate, succinate and itaconate; glial cells decode these metabolic cues and convert them into inflammatory or pro-resolving programmes; and the resulting cytokines, growth factors and physicochemical changes reshape nociceptive circuits in the dorsal root ganglion (DRG) and spinal dorsal horn. The same relay is further sculpted by spatial and temporal heterogeneity: metabolites accumulate in discrete microdomains, propagate as “metabolic waves” through glial networks and leave longer-lasting traces in the form of metabolic memory and circadian modulation. At the clinical level, these processes manifest as measurable metabolic pain phenotypes that correlate with symptom burden and may predict treatment response. Figure 1 outlines the core architecture of the metabolite–neuro–immune relay model proposed in this Review.

We first summarise how pathological metabolic states arise in pain-relevant tissues and generate distinct lactate, succinate and itaconate signatures. We then discuss how microglia and astrocytes decode these signals and relay them to nociceptors and spinal circuits, before considering how spatial and temporal organisation of metabolite signalling shapes the evolution from acute to chronic pain. We next examine emerging evidence for metabolite-based pain phenotypes in patients and outline how these signatures can inform mechanism-based stratification. Finally, we review therapeutic strategies that aim to reset the metabolite–neuro–immune relay, ranging from metabolic enzyme inhibition and targeted scavenging to nanodelivery and synthetic biology. By explicitly framing chronic pain as a disorder of metabolically encoded neuroimmune plasticity, we aim to provide a working model that connects basic mechanisms with clinical translation and highlights concrete, testable targets for future intervention.

## 2 Upstream metabolic perturbations: generating lactate, succinate and itaconate

Chronic pain seldom arises from a single lesion; it is sustained by durable shifts in cellular metabolism (Li et al., 2024a). Injury, chemotherapy and systemic inflammation reprogramme glia and immune cells toward glycolysis and altered tricarboxylic acid (TCA) flux, producing characteristic patterns of lactate, succinate and itaconate (Kong et al., 2022). Here we focus on how these metabolites are generated in astrocytes, microglia and peripheral immune cells, establishing the input layer of the metabolite–neuro–immune relay that later reshapes nociceptive circuits. Table 1 provides a concise overview of the major sources and core signalling logic of lactate, succinate and itaconate that seed the metabolite–neuro–immune relay.

**TABLE 1:** Comparative features of lactate, succinate, and itaconate in chronic pain.

Dimension	Lactate	Succinate	Itaconate
Dominant source	Astrocytes (major CNS source); activated myeloid cells (context-dependent)	Immune/glial TCA stress	IRG1^+^ microglia/macrophages
Key sensing routes	MCT1/4; HCAR1 (evidence evolving in microglia)	SUCNR1	SDH inhibition; KEAP1–NRF2
Main microenvironmental effect	Acidosis; fuel/signal	HIF-1α/ROS bias	Redox/anti-inflammatory bias
Typical circuit outcome	Supports sensitization	Amplifies nociceptor gain	Facilitates resolution
Conceptual role in relay	Context-dependent “amplifier/modulator”	Predominant “go” signal	Predominant “stop/brake” signal
Human signal	Elevated in CSS/FM cohorts (CSF/brain; emerging)	Elevated in FM cohorts and OA-related tissues (emerging)	Direct clinical pain data limited

Statements summarise convergent trends across representative inflammatory and neuropathic models; human evidence remains emerging and largely associative.

### 2.1 Lactate: pathological glycolysis and astrocyte–microglia shuttling

Under pathological conditions such as peripheral nerve injury or chemotherapy-induced neuropathy, astrocytes in the central nervous system (CNS) shift toward glycolysis and release excess lactate, raising extracellular concentrations (Wen et al., 2025). Lactate is then transferred to neighbouring cells via monocarboxylate transporters (MCTs). Microglia import lactate mainly through monocarboxylate transporters MCT4 and MCT1; in lipopolysaccharide-activated microglia, MCT1 expression and glycolytic flux increase, whereas MCT1 knockdown suppresses glycolysis and reduces induction of IL-1β and TNF-α (Kong et al., 2019; Škandík et al., 2025). Similar MCT-dependent effects have been described in macrophages, in which lactate amplifies toll-like receptor–NF-κB signalling (Yang et al., 2022; Lauterbach et al., 2019; Chen et al., 2025). Together, these observations indicate that lactate uptake via MCTs reinforces a pro-inflammatory metabolic state in myeloid cells and that elevated spinal lactate can act as an upstream amplifier of neuroinflammation.

*In vivo*, disrupting the astrocyte–neuron lactate shuttle modifies pain hypersensitivity. Selective activation of spinal astrocytes provokes persistent mechanical hyperalgesia, whereas blocking astrocytic lactate export with the broad MCT inhibitor α-cyano-4-hydroxycinnamate (4-CIN) reverses hyperalgesia and dampens glial reactivity (Miyamoto et al., 2019). Conversely, intrathecal L-lactate lowers pain thresholds and increases neuronal activity markers; these effects are abolished by MCT inhibition or interference with lactate metabolism (Miyamoto et al., 2019; Kambe et al., 2022). Genetic or pharmacological strategies that reduce lactate production or transport similarly lower spinal cytokine levels and attenuate nociceptive sensitization (Cheng H. J. et al., 2024; He et al., 2019). These data support a metabolic feed-forward loop in which excess astrocytic lactate sustains pain through persistent neuron–glia interactions.

Beyond its role as an energy substrate, lactate also acts as a signalling molecule. High glycolytic flux in activated microglia drives histone lactylation, which promotes transcription of inflammatory genes, whereas glycolysis inhibition reduces lactate, diminishes histone lactylation and attenuates neuroinflammation (Qin et al., 2025; He et al., 2024; Cheng et al., 2021). Lactate additionally signals via hydroxycarboxylic acid receptor 1 (HCAR1, also known as G protein-coupled receptor 81), expressed in neurons and astrocytes, where Gi-coupled signalling dampens excitability and neurotransmitter release, suggesting a potential feedback brake on network activity (de Castro Abrantes et al., 2019). By contrast, the relevance of HCAR1 to microglial lactate sensing remains unclear, and some data even point to anti-inflammatory effects of lactate–HCAR1 signalling in myeloid cells (Ma et al., 2020; Peters et al., 2022; Pan et al., 2025). Disentangling these concentration- and cell type-dependent actions remains a key challenge, but the overall picture places lactate as a central neuroimmune signal linking astrocyte metabolism to microglial activation and neuronal hyperexcitability.

### 2.2 Succinate: SUCNR1 signalling and metabolic–epigenetic crosstalk

Succinate, a core TCA intermediate, has likewise emerged as a modulator of neuroinflammation in chronic pain (Ruan et al., 2021). In microglia, elevated intracellular succinate—modelled with cell-permeable analogues—can alter inflammatory polarisation and mitochondrial dynamics, whereas extracellular succinate evokes calcium transients in subsets of astrocytes, linking metabolic stress to neuron–glia communication (Sangineto et al., 2023; Molnár et al., 2011).

A major route for extracellular signalling is SUCNR1 (also known as G protein-coupled receptor 91) (Trauelsen et al., 2021). In inflammatory settings, macrophage-derived succinate activates SUCNR1 on neural stem cells, glia and vascular endothelium, triggering p38 mitogen-activated protein kinase cascades and prostaglandin E_2_ release, and in some contexts promoting clearance of extracellular succinate and reprogramming macrophages toward a less inflammatory phenotype (Peruzzotti-Jametti et al., 2018; Keiran et al., 2019; Wu, 2023). In peripheral inflammatory pain models, excessive succinate and SUCNR1 activation stabilise HIF-1α, promote NLRP3 inflammasome assembly and enhance IL-1β release; pharmacological interruption of the succinate/SUCNR1–HIF-1α–NLRP3 axis reduces IL-1β, limits transient receptor potential vanilloid 1 (TRPV1) upregulation in sensory neurons and attenuates nociceptive hypersensitivity (Ruan et al., 2021). Endothelial SUCNR1 signalling can similarly induce HIF-1α and IL-1β, further amplifying inflammatory cascades (Xu et al., 2022; Huang et al., 2024).

Translation is complicated by both expression patterns and receptor-independent actions. In rodents, Sucnr1 is most abundant in the retina and relatively low in other CNS regions, whereas in humans SUCNR1 is broadly expressed in immune populations such as immature dendritic cells and macrophages and in non-immune cells including vascular smooth muscle and renal tubular epithelium (Favret et al., 2013; Keiran et al., 2019; Gilissen et al., 2016). Succinate accumulation also promotes histone succinylation and inhibits α-ketoglutarate-dependent dioxygenases such as ten-eleven translocation (TET) DNA demethylases, thereby reshaping the epigenetic landscape during inflammation (Xiao et al., 2012; Wang et al., 2017; López-Moyado et al., 2024). These dual modes of action—SUCNR1-dependent and metabolic–epigenetic—underscore succinate’s broad influence on immune tone and highlight why isolating a single “druggable” node along this pathway is challenging.

Finally, succinate’s effects are highly context dependent. Although often portrayed as a pro-inflammatory danger signal, SUCNR1 activation in adipose-tissue macrophages can induce anti-inflammatory transcriptional programmes and ameliorate metabolic disease, illustrating tissue-specific homeostatic roles. Human studies in inflammatory conditions report elevated circulating succinate that correlates with IL-1β, consistent with its capacity to drive cytokine production (Gilissen et al., 2016; Xu et al., 2022; Yan et al., 2022). For chronic pain, these observations imply that targeting succinate signalling will need to account for timing, tissue context and patient-specific immune states, and that human-derived tissues, organoids and *ex vivo* models will be essential to define when succinate acts as a driver of pro-nociceptive inflammation and when it contributes to adaptive resolution.

### 2.3 Itaconate: IRG1/NRF2-mediated resolution of neuroinflammation

Itaconate is generated from cis-aconitate by IRG1 (also known as aconitate decarboxylase 1, ACOD1) and has emerged as a key immunometabolite linking TCA cycle activity to the active resolution of inflammation (Lampropoulou et al., 2016). Produced mainly by activated macrophages and microglia, itaconate modulates mitochondrial redox balance and gene expression and is now viewed as a signalling hub rather than a passive TCA by-product. A core mechanism is inhibition of succinate dehydrogenase (SDH, complex II), which limits succinate oxidation, reduces electron transport and lowers mitochondrial reactive oxygen species (ROS) generation. In parallel, itaconate alkylates Kelch-like ECH-associated protein 1 (KEAP1), stabilising NRF2 and inducing an antioxidant, cytoprotective transcriptional programme. Through coordinated control of SDH/ROS and KEAP1/NRF2, itaconate imposes a broadly anti-inflammatory tone in neuroinflammatory settings (Mills et al., 2018; Liu et al., 2018; Qian et al., 2024).

Downstream, itaconate reshapes both inflammasome activity and cytokine output. Itaconate and cell-permeable derivatives such as 4-octyl itaconate (4-OI) covalently modify nucleotide-binding oligomerization domain, leucine-rich repeat and pyrin domain-containing protein 3 (NLRP3), preventing assembly of the active inflammasome. In Irg1-deficient macrophages, NLRP3 activation and IL-1β release are exaggerated, whereas pharmacological augmentation of itaconate reduces NLRP3-driven inflammation *in vivo* and lowers spinal IL-1β in inflammatory pain models in parallel with reduced behavioural hypersensitivity (Hooftman et al., 2020; Cai et al., 2023; Lin et al., 2022). In microglia and macrophages, increased itaconate also boosts IL-10 via NRF2-dependent pathways, promotes a reparative, M2-like phenotype and suppresses TNF-α and other pro-inflammatory mediators. In rodent models of persistent pain, derivatives such as dimethyl itaconate and 4-OI attenuate neuroinflammation and mechanical and thermal hypersensitivity, and the analgesic effect of 4-OI is lost in IL-10-deficient mice, supporting an IL-10/IRG1 axis that couples inflammasome restraint to pro-resolving cytokine profiles (Sun et al., 2022; Ren et al., 2022; Lin et al., 2022).

Emerging data further suggest paracrine neuroprotective actions. In neuroinflammatory models, microglia in defined brain regions upregulate IRG1 and itaconate; disrupting this pathway worsens neuronal dysfunction, whereas 4-OI restores neuronal activity and improves behavioural outcomes (Liu et al., 2025; Ni et al., 2022). Conditioned media from itaconate-treated microglia protect cultured neurons from toxic injury, consistent with autocrine and paracrine actions of microglia-derived itaconate that buffer nearby neurons against inflammatory stress (Xia et al., 2023; Ni et al., 2022). Although clinical data are still sparse and largely derived from rodent or *in vitro* systems, these converging findings position the IRG1–itaconate–NRF2 axis as an endogenous brake within the metabolite–neuro–immune relay and a rational target for mechanism-based analgesic strategies.

In chronic pain, astrocytes and microglia undergo metabolic reprogramming, resulting in the abnormal production of lactate, succinate, and itaconate. These three metabolites form a dynamic metabolic network that synergistically regulates neuroinflammation. Lactate initiates inflammatory responses, while succinate inherits and amplifies pro-inflammatory signals, collectively contributing to nociceptive sensitization. In contrast, itaconate moderately inhibits excessive inflammation in the early stage; in the later stage, it acts as a negative feedback factor, antagonizing the pro-inflammatory effects of the other two metabolites by inhibiting succinate dehydrogenase (SDH) and stabilizing nuclear factor E2-related factor 2 (NRF2), thereby facilitating inflammation resolution. These three metabolites interact with and restrict each other, and their imbalance can induce persistent inflammation and sustain chronic pain. Thus, restoring the balance of this metabolic network may offer a novel strategy for chronic pain management.

## 3 Metabolite–neuro–immune relay: from immune activation to neuronal sensitisation

The preceding section outlined how upstream insults reshape cellular metabolism to produce distinct lactate, succinate and itaconate signatures. These changes become clinically relevant only when they are decoded by glia and immune cells and passed on to nociceptive circuits. In this section, we examine this intermediate step: how microglia and astrocytes sense metabolite shifts, engage inflammatory and plasticity pathways, and translate metabolic stress into persistent neuronal hyperexcitability. Section 3.1 considers glia as the central decoding hub of the relay, whereas Sections 3.2, 3.3 focus on how succinate and itaconate, respectively, implement “go” and “stop” signals at the level of nociceptors and spinal circuits.

### 3.1 Glial decoding of metabolic stress

Chronic pain does not arise from neurons in isolation. Microglia, astrocytes and infiltrating immune cells continuously sample their metabolic milieu, translate metabolic stress into inflammatory programmes and relay this information to nociceptive circuits. In glia, shifts toward glycolysis and disrupted mitochondrial function are tightly coupled to activation of HIF-1α, NF-κB and NLRP3. These pathways convert changes in lactate, succinate and redox balance into cytokine release, ROS production and altered synaptic support, forming the core of a metabolite–neuro–immune relay that links upstream metabolic perturbations to neuronal sensitization.

Microglia act as a primary decoding hub. In neuropathic and inflammatory pain models, spinal microglia reprogramme from oxidative phosphorylation to aerobic glycolysis, with enhanced HIF-1α signalling (Kong et al., 2023; Li et al., 2024b; Zhang et al., 2022). This state favours production of IL-1β, tumour necrosis factor and brain-derived neurotrophic factor (BDNF), which together strengthen excitatory synapses, weaken inhibitory tone and remodel dorsal horn circuitry (Qiu et al., 2023; Atta et al., 2023). Lactate and succinate are key inputs to this programme, stabilizing HIF-1α and promoting NLRP3 activation in myeloid cells, whereas resolution-phase metabolites such as itaconate can impose an opposing, NRF2-dependent anti-inflammatory state. Thus, rather than being passive by-products, these metabolites help set the inflammatory “tone” of microglia and bias them toward either amplifying or damping nociceptive signalling.

Glial cells exhibit striking cell-type specificity in decoding metabolic stress, with astrocytes, microglia, and oligodendrocytes displaying divergent sensing mechanisms and functional responses to lactate, succinate, and itaconate. Microglia act as primary sentinels for lactate and succinate: they take up lactate via MCT1/4, detect succinate through SUCNR1, and activate HIF-1α and NLRP3 signalling cascades to drive proinflammatory programmes (Saraiva et al., 2018; Zhang et al., 2024). In contrast, astrocytes are central to lactate metabolism regulation: they generate lactate via glycolysis, propagate metabolic signals through MCT-dependent shuttle systems, and sense extracellular succinate via SUCNR1 to elicit calcium transients that modulate neuron–glia communication. Although understudied in chronic pain metabolism, oligodendrocytes take up lactate via specialised transporters to meet myelin synthesis’ high energy demands. Furthermore, itaconate attenuates oligodendrocyte oxidative stress by activating the NRF2 pathway, indirectly preserving myelin integrity and neural conduction (Liu et al., 2025). Collectively, this cell-type-specific decoding forms the cellular framework for translating metabolic stress signals into neuroinflammation, enabling precise signal discrimination and context-appropriate glial responses. Importantly, however, other relevant cell types—including neurons, endothelial cells, pericytes, and meningeal immune populations—also contribute to the chronic pain metabolic microenvironment and thus warrant systematic future investigation. Astrocytes operate at the interface between metabolism and network activity. Persistent nociceptive input drives astrocytic glycolysis and glycogen turnover, increasing lactate export to active neurons via monocarboxylate transporters (He et al., 2019). Acutely, this astrocyte–neuron lactate shuttle supports synaptic transmission; in chronic pain, sustained glycolytic flux and local acidosis transform this support into a feed-forward driver of central sensitization (Ma et al., 2024; Hu et al., 2024). Genetic disruption of astrocyte glycogen metabolism shortens the maintenance phase, but not the initiation, of pain *in vivo*, indicating that altered astrocytic energy reserves help stabilise, rather than trigger, hypersensitivity (Liu et al., 2023; Cheng et al., 2020). In parallel, astrocytes release glutamate and ATP as gliotransmitters, further tuning synaptic strength and excitability within dorsal horn networks.

At the circuit level, the combined actions of microglia and astrocytes couple metabolic state to neuronal output. Pro-inflammatory glia release IL-1β, BDNF and complement components that reshape synaptic connectivity, while changes in extracellular pH, ATP and metabolite concentrations directly modulate ion channels and receptors on nociceptors and dorsal horn neurons (Boakye et al., 2021; Chu J. et al., 2023). In this framework, lactate and succinate function predominantly as “go” signals that favour a high-gain, hyperexcitable network state, whereas itaconate and related resolution-phase metabolites provide “stop” signals that bias glia toward reparative phenotypes. The following sections examine how specific metabolite–receptor axes—most notably succinate–SUCNR1 and the IRG1–itaconate–NRF2 pathway—implement this relay at the level of primary afferents and spinal circuits.

### 3.2 Succinate-driven neuronal hyperexcitability

Within the metabolite–neuro–immune relay, succinate functions as a prototypical immune-to-neuron signal. In chronic inflammatory and neuropathic pain, activated macrophages and microglia accumulate succinate and release it into the extracellular space, where it acts as a local distress cue (Littlewood-Evans et al., 2016; Wang et al., 2021). Ligand binding engages G-protein–coupled cascades, including Gq–phospholipase Cβ signalling, elevates intracellular calcium and activates calcium-dependent kinases and transcription factors. These pathways increase the expression and sensitization of ion channels such as TRPV1, thereby lowering activation thresholds and promoting hyperexcitability of primary afferents.

Preclinical models converge on this axis. Inflammatory joint and soft-tissue injury paradigms demonstrate that tissue succinate levels increase in parallel with pain-related behaviors, and pharmacological or genetic inhibition of SUCNR1 attenuates local inflammation and mechanical hypersensitivity (Littlewood-Evans et al., 2016; Guo et al., 2022). The concentrations required for effective SUCNR1 activation are substantially higher than those associated with normal TCA cycle flux, supporting the notion that, under inflammatory or pain conditions, succinate functions not merely as a metabolic intermediate but as a paracrine “danger” signal (Wu, 2023; Peruzzotti-Jametti et al., 2018). In addition, excess intracellular succinate can inhibit α-ketoglutarate-dependent dioxygenases and promote histone succinylation in sensory neurons, loosening chromatin and facilitating transcriptional programmes linked to persistent sensitization as supported by recent findings showing increased succinylation and related acylation in DRG neurons after nerve injury, where KAT2A-mediated histone succinylation enhances pro-inflammatory gene expression (Zou et al., 2022; Chen et al., 2024). Together, these receptor-dependent and epigenetic actions position succinate as a key link between immune metabolic stress and long-lasting nociceptor plasticity, even though species differences in SUCNR1 distribution will need to be considered when translating SUCNR1-targeted strategies to the clinic.

### 3.3 Immunometabolic feedback: itaconate as a brake on neuroimmune activation

Itaconate occupies the opposite pole of the relay, acting as an endogenous brake on metabolite-driven neuroinflammation. In activated microglia and macrophages, IRG1–dependent itaconate synthesis inhibits succinate dehydrogenase and activates NRF2-driven antioxidant programmes, counterbalancing the glycolytic, HIF-1α- and NLRP3-biased states described above (Mills et al., 2018; Lampropoulou et al., 2016; Swain et al., 2020; Ni et al., 2022). Within spinal pain circuits, this immunometabolic shift dampens microglial activation and cytokine release and indirectly protects neurons from inflammation-induced hyperexcitability. Cell-permeable itaconate derivatives such as 4-OI and dimethyl itaconate attenuate neuroinflammation and mechanical and thermal hypersensitivity in preclinical models, in part by suppressing NLRP3 inflammasome activation and IL-1β production and by increasing IL-10 (Hooftman et al., 2020; Lin et al., 2022; Darvish et al., 2022; Zhao et al., 2025). The loss of analgesic efficacy in IL-10-deficient mice supports an IL-10–IRG1 feedback loop through which itaconate reinforces pro-resolving cytokine profiles (Sun et al., 2022; Hooftman et al., 2020).

Crucially, this brake is engaged in a bidirectional manner. Signals arising from damaged or inflamed neural environments, rather than from neuronal hypoactivity *per se*, can upregulate IRG1 expression in neighbouring microglia, enhancing itaconate production as an adaptive counter-regulatory response (Ni et al., 2022; Wang et al., 2025; Swain et al., 2020). In neuroinflammatory models, genetic or pharmacological disruption of the microglial IRG1–itaconate axis exacerbates neuronal dysfunction, whereas restoration with cell-permeable itaconate derivatives preserves neuronal activity and ameliorates motor and cognitive deficits (Ni et al., 2022; Kuo et al., 2021; Qian et al., 2024; Kong et al., 2024; Zhao et al., 2025; Chang et al., 2024). Although human data remain limited, these observations support a two-way circuit in which neuronal injury triggers a metabolic anti-inflammatory response that feeds back to stabilise vulnerable networks. Against the backdrop of lactate- and succinate-driven excitation, the IRG1–itaconate–NRF2 pathway thus emerges as a central endogenous counterweight and a promising target for therapies aiming to rebalance neuroimmune signalling without broadly suppressing host defence.

In summary, several key controversies persist regarding the metabolic network composed of lactate, succinate, and itaconate: the dual and contradictory pro-inflammatory and anti-inflammatory effects of lactate mediated by HCAR1; the species-specific distribution of SUCNR1, which poses a constraint on the clinical translation of succinate-targeted analgesic strategies; and the unclear cell-type-specific regulatory mechanisms underlying itaconate’s anti-inflammatory actions. These unresolved controversies suggest that future research should integrate diverse cell subtypes and species models, delve into the cross-regulatory patterns of the three metabolites’ signaling pathways, and validate these findings using human chronic pain samples. This approach will provide precise evidence for the development of targeted analgesic therapies focusing on the “metabolite-neuro-immune” relay.

## 4 Spatial and temporal modulation of metabolite signalling in chronic pain

**FIGURE 2:**
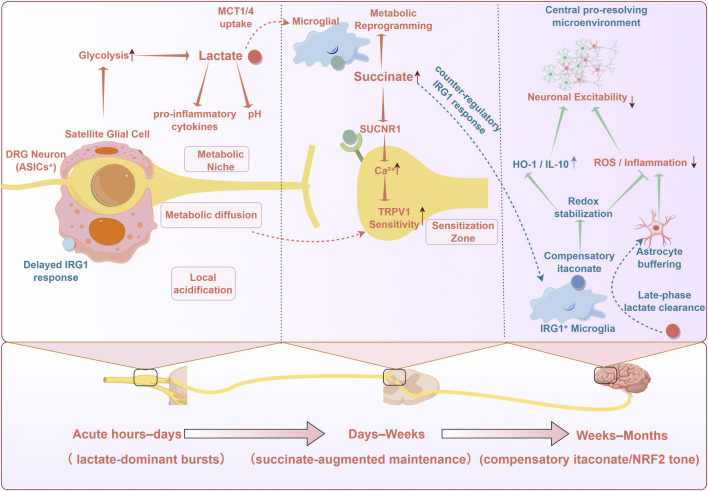
Time-resolved, regionally structured trajectory of metabolite signalling during pain chronification. Lactate-, succinate- and itaconate-biased microenvironments are depicted as phase-resolved modulators of neuroimmune coupling across the DRG–spinal–brain axis. Acute lactate-dominant bursts (hours–days) associated with local acidification can favour early sensitising conditions. Succinate-linked maintenance (days–weeks) engages SUCNR1 on microglia and nociceptors to promote Ca^2+^-dependent signalling and increased TRPV1 sensitivity within a sensitisation zone. A delayed IRG1–itaconate response (weeks–months) is proposed to reinforce a central pro-resolving state, with NRF2-linked redox stabilisation, increased HO-1/IL-10 tone, astrocyte buffering and late-phase lactate clearance limiting ROS/inflammation and neuronal excitability. Red arrows indicate pro-inflammatory or pro-sensitising effects; green arrows indicate anti-inflammatory or resolution-biased effects.

Signalling metabolites do not bathe nociceptive pathways uniformly. Instead, lactate, succinate and itaconate form spatially and temporally structured patterns that shape how DRG neurons, spinal circuits and glia respond to injury. Local metabolic niches in the DRG and spinal dorsal horn, together with dynamic shifts from transient metabolite bursts to stable metabolic reprogramming, determine whether the metabolite–neuro–immune relay resolves or locks into a chronic state. Here we focus on how space and time modulate the mechanisms outlined above, rather than revisiting their detailed biochemistry. These spatial–temporal features of the metabolite–neuro–immune relay are summarised in Figure 2.

### 4.1 Metabolic microdomains and spatial metabolomics: tools and challenges

Evidence from animal and human tissues indicates that metabolic signalling in chronic pain is anatomically structured rather than diffuse. Lactate and succinate accumulate in discrete microdomains, particularly within the DRG and spinal dorsal horn, where they influence microglial and astrocytic activation as well as nociceptor sensitivity (Miao et al., 2023). These “metabolic niches”, shaped by inflammation, perfusion and local cell composition, can sustain self-reinforcing neuroinflammatory loops.

Within such niches, relatively modest biochemical shifts can have disproportionate effects on local excitability. Inflammatory hypoxia enhances astrocytic glycolysis and lactate export, and the resulting acidification activates acid-sensing ion channels on nociceptors while lactate shuttling through monocarboxylate transporters sustains pro-inflammatory microglial signalling, partly via toll-like receptor–dependent pathways. Succinate enrichment in perineuronal glia engages local SUCNR1–mediated signalling that amplifies neuronal excitability and glial inflammation, while at later stages, macrophages and microglia in the same regions induce IRG1 and generate itaconate, which activates NRF2 and IL-10 programmes, forming a local negative feedback on lactate- and succinate-driven neuroinflammation (Kong et al., 2025; Ren et al., 2022; Zhao et al., 2025; Rahimi et al., 2024; Belo et al., 2023; Ohm et al., 2024). The balance between these pro-inflammatory and pro-resolving signals within each microdomain helps determine whether circuits normalise or remain sensitised.

Metabolic cues can propagate beyond their site of origin. Astrocytic mitochondrial calcium transients, arising from localized mitochondrial permeability transition events, have been shown to shape microdomain metabolic activity and support glycolysis (Agarwal et al., 2017). These calcium-driven metabolic responses can generate localized NADH and glycolytic oscillations within astrocytic networks, and the resulting lactate produced by aerobic glycolysis may diffuse through gap-junction-coupled astrocytes to redistribute energy substrates (Ahmadpour et al., 2021; Cooper et al., 2020; Murphy-Royal et al., 2020). Other metabolites, including succinate and adenosine triphosphate, can diffuse through extracellular spaces or move along axons, acting as spatially encoded signals along the nociceptive pathway. In this broader framework, metabolic flux becomes a communication channel that conveys information about neural activity and tissue stress across the pain circuitry.

Recent advances in spatial metabolomics have begun to visualise these microdomains and gradients directly. Matrix-assisted laser desorption/ionization mass spectrometry imaging (MALDI-MSI) and desorption electrospray ionization mass spectrometry imaging (DESI-MSI) have been applied to pain-relevant tissues to map metabolites *in situ* (Murphy-Royal et al., 2020). These approaches reveal metabolite-enriched regions in spinal cord and DRG that coincide with zones of glial reactivity and structural remodelling (McKinnon et al., 2024). MALDI-MSI offers near-cellular spatial resolution, enabling fine-grained mapping of DRG neuron–satellite cell units or dorsal horn laminae, whereas DESI-MSI provides higher throughput and excellent preservation of tissue architecture, allowing rapid surveys of larger regions or multiple segments (McKinnon et al., 2024; Bian et al., 2021; Chen et al., 2019). Together, they provide complementary “zoomed-in” and “landscape-level” views of metabolic organisation.

Important limitations remain. Most spatial metabolomics datasets derive from *ex vivo* sections and capture only static snapshots of metabolic states. Live imaging of specific metabolites in the intact nervous system is technically demanding, constrained by tissue penetration, spatial resolution and the ability to distinguish individual metabolites in real time over the course of pain chronification. Moreover, metabolite maps are often generated without concurrent readouts of neuronal activity or immune phenotypes in the same regions, complicating causal inference: a lactate hotspot, for example, may precede, drive or merely accompany local glial activation and spiking (Reid et al., 2025; Tang et al., 2021).

Future work will require multimodal, time-resolved approaches that integrate spatial metabolomics with functional and phenotypic data. Combining metabolic imaging with calcium imaging or electrophysiology, alongside spatial immune profiling (for example, co-immunostaining or spatial transcriptomics), would allow direct linkage of metabolic changes to neuronal excitability and glial states within the same microdomains. Longitudinal sampling after injury could support phase-resolved models of how acute metabolic bursts evolve into distributed maintenance signals. Such integrated maps of metabolite–neuro–immune coupling may reveal pathogenic metabolic waves that herald pain flares or protective niches associated with resilience, and in turn identify targets for interventions that either disrupt pathological signalling or reinforce beneficial microdomain activity.

### 4.2 Temporal dynamics of metabolite signalling: from transient bursts to sustained reprogramming

Beyond spatial heterogeneity, the metabolite–neuro–immune relay is highly dynamic in time. Acute noxious input can trigger rapid, short-lived surges in metabolic activity: activated glia switch to glycolysis, generating local lactate spikes that diffuse to adjacent neurons within minutes. Exogenous lactate, applied on a similar timescale, can acutely facilitate calcium influx and increase neuronal excitability. These fast transients probably propagate through gap-junction-coupled astrocytic networks as combined calcium–metabolic waves.

With repeated or sustained stimulation, however, these transient bursts consolidate into more durable metabolic disturbances. Pro-inflammatory microglia and astrocytes adopt a glycolytic, high-lactate state and accumulate TCA intermediates such as succinate, engaging hypoxia-inducible and inflammasome pathways described above and driving a self-sustaining loop of cytokine release and neuroinflammation. Chronically activated astrocytes increase glycolytic flux, upregulate monocarboxylate transporters and augment glycogen stores in the spinal cord. *In vivo*, deletion of the glycogen-targeting protein PTG in astrocytes leaves early nociceptive responses intact but abbreviates the duration of central sensitization, indicating that augmented energy reserves and persistent glycolysis stabilise rather than initiate chronic pain states (Marty-Lombardi et al., 2024). Consistently, genetic or pharmacological interference with key glycolytic regulators reduces spinal lactate accumulation and attenuates hyperalgesia in models of diabetic neuropathy and nerve injury, supporting the view that long-term metabolic reprogramming encodes a form of “metabolic memory” in nociceptive circuits (Cheng H. J. et al., 2024; Li et al., 2024b; Hua et al., 2024).

On an even broader timescale, circadian rhythms modulate metabolite signalling and pain sensitivity. Many inflammatory and neuropathic conditions show characteristic diurnal oscillations in symptom severity and inflammatory–metabolic markers, paralleling circadian variation in immune activity, stress hormones and cellular metabolism. Disruption of core clock genes lowers pain thresholds and prolongs pain after injury, highlighting their regulatory links with metabolic gene networks that influence the induction and resolution phases of chronic pain (Kim et al., 2020; Zhang et al., 2019; Warfield et al., 2021; Chu Y. et al., 2023).

These temporal layers—from millisecond lactate–calcium transients to weeks-long metabolic reprogramming and circadian oscillations—have direct therapeutic implications. Chronotherapy, in which anti-inflammatory or metabolism-modulating drugs are administered at phases of maximal vulnerability or drug sensitivity, has already improved outcomes in disorders such as rheumatoid arthritis (Ursini et al., 2021). Applying similar principles to metabolically targeted analgesics may offer a way to “reset” maladaptive metabolic states, weaken embedded metabolic memory and widen the window for durable pain relief.

In addition to controversies surrounding mechanisms at the molecular level, technological limitations also hinder a deeper understanding of metabolic network dynamics. The key unresolved controversies currently include: static snapshots of spatial metabolomics cannot clearly establish the causal relationship between metabolite microdomain enrichment and glial activation; existing live imaging techniques cannot track the dynamics of metabolites in real time within the entire nervous system; and the molecular mechanisms underlying the circadian regulation of metabolite signals remain unclear (McKinnon et al., 2024; Reid et al., 2025; Kim et al., 2020). Future research should combine multimodal spatiotemporal imaging with longitudinal disease models, integrate multidimensional data, and elucidate the association between metabolite spatiotemporal dynamics and chronic pain, thereby providing a theoretical basis for temporally dependent targeted analgesic strategies.

## 5 Metabolic pain phenotypes and clinical translation

Work on lactate, succinate and itaconate has moved signalling metabolites from mechanistic curiosities to potential clinical tools. Across chronic pain syndromes, distinct constellations of these and other metabolites increasingly define metabolic pain phenotypes that correlate with symptom burden and may help predict treatment response. In this section, we highlight how such signatures manifest in patients and what they imply for diagnosis, stratification and mechanism-based intervention, without repeating mechanistic detail covered in earlier chapters.

### 5.1 Metabolic pain phenotypes and clinical implications

Across conditions such as fibromyalgia, osteoarthritis and chemotherapy-induced neuropathy, untargeted metabolomics consistently distinguishes patients from healthy controls by coordinated changes in energy-related metabolites, amino acids and lipids. Fibromyalgia cohorts show elevated lactate and succinate, consistent with glycolytic and mitochondrial stress and altered microbiome–host metabolism (Piras et al., 2022; Macchi et al., 2024; Ramírez-Tejero et al., 2023). Chemotherapy-induced neuropathy is characterized by disrupted glucose utilization and altered TCA cycle intermediates, reflecting mitochondrial metabolic imbalance (Zhao et al., 2024; Winter et al., 2023; Behl et al., 2023). In several studies, small panels that include succinate together with metabolites such as taurine and creatine discriminate patients from controls with high diagnostic accuracy and track pain and fatigue scores over time.

These patterns reinforce the view that chronic pain represents a state of systemic metabolic dysregulation tightly coupled to immune activation, rather than a purely regional neural disorder. Clinically, this creates two immediate opportunities. First, metabolite profiling could support stratification by pain subtype or stage and provide objective markers of trajectories, particularly around the transition from acute to chronic pain. Second, recurrent hubs in these signatures—such as sustained glycolytic reprogramming or the succinate–inflammasome axis—offer concrete entry points for mechanism-based therapy and rational patient selection for metabolically targeted interventions.

### 5.2 Succinate: clinical and translational evidence for pain amplification

Clinical and translational data converge on succinate as both a biomarker and a driver of pain amplification. In fibromyalgia, urinary succinate is consistently higher than in healthy controls and contributes to diagnostic metabolite panels that correlate with pain intensity and fatigue, in keeping with a shift toward a pro-inflammatory metabolic state (Malatji et al., 2017; Fineschi et al., 2022). Similar elevations have been reported in other inflammatory conditions, often paralleling IL-1β levels, consistent with engagement of the SUCNR1–inflammasome axis described earlier.

Local tissue studies provide complementary evidence. In metabolically driven osteoarthritis, cartilage from affected joints shows succinate accumulation together with upregulation of SUCNR1 on chondrocytes, and in injury models succinate build-up increases SUCNR1 and inflammasome expression, fuels IL-1β release, and drives mechanical hypersensitivity (Shen et al., 2019; Ni et al., 2019). Experimental interventions that lower succinate or block SUCNR1 reduce joint inflammation, spinal neuroinflammation and nociceptive behaviours (Guo et al., 2022; Nunns et al., 2022). Taken together, these findings position succinate as a neuromodulatory metabolite that links cellular metabolic distress to amplified pain signalling and highlight the succinate pathway as a promising target for metabolically informed analgesic strategies.

### 5.3 Itaconate: an endogenous anti-inflammatory brake on pain

Itaconate, generated by IRG1 in activated macrophages and microglia, counterbalances many of the inflammatory processes driven by lactate and succinate. As outlined above, itaconate inhibits succinate dehydrogenase, activates NRF2 and constrains NLRP3 inflammasome activity, collectively shifting cytokine profiles away from IL-1β and TNF-α toward IL-10. In the context of chronic pain, this constellation of effects is best viewed as an intrinsic “brake” within the metabolite–neuro–immune relay, rather than as an isolated anti-inflammatory pathway.

Direct data on itaconate in human pain cohorts are still scarce, but reduced circulating itaconate has been reported in several chronic inflammatory disorders, consistent with a weakened endogenous resolution response. By contrast, preclinical pain models consistently show that dimethyl itaconate and related derivatives alleviate mechanical and thermal hypersensitivity while dampening neuroinflammation in dorsal root ganglia and spinal cord. Loss of analgesic efficacy when NRF2 is inhibited or IRG1 is disrupted confirms pathway specificity (Ren et al., 2022; Sun et al., 2022). Together, these findings support itaconate as both a candidate biomarker of intact resolution capacity and a tractable target for mechanism-based analgesic development.

### 5.4 Lactate dysregulation and pain sensitisation

Lactate is now recognised as a neuromodulatory metabolite whose dysregulation mirrors, and may help sustain, chronic pain. Centrally, patients with fibromyalgia and related central sensitivity syndromes show elevated ventricular cerebrospinal fluid lactate on magnetic resonance spectroscopy compared with healthy controls, pointing to a shared brain “metabolic stress” phenotype rather than a disease-specific signature (Natelson et al., 2017; Natelson et al., 2015). Peripherally, microdialysis of painful muscles such as the trapezius reveals higher interstitial lactate and pyruvate at rest, and these elevations correlate with reduced pressure-pain thresholds (Gerdle et al., 2010; Gerdle et al., 2020).

These clinical patterns dovetail with experimental evidence that lactate can potentiate acid-sensing ion channels, promote ROS generation and modulate astrocyte–neuron shuttling, thereby lowering the activation threshold of nociceptive pathways. Across syndromes, elevated lactate in brain, muscle and blood tracks with hyperalgesia and symptom burden, reinforcing the view that lactate is not inert “metabolic waste” but a clinically relevant signal within sensitised networks (Gerdle et al., 2020; Clos-Garcia et al., 2019). More precise definition of lactate-centred metabolic endotypes—using human tissues, *in vivo* imaging and advanced cellular models—may help explain persistent sensitisation in disorders such as fibromyalgia and identify metabolic nodes that can be targeted in a rational, phenotype-guided manner.

In summary, regarding the roles of itaconate, lactate, and succinate in chronic pain, several key controversies in current research urgently require clarification. The expression and regulatory mechanisms of itaconate in human chronic pain cohorts are unclear, and its epigenetic targets (e.g., TET2) mediating anti-inflammatory and analgesic effects have not been fully validated. Additionally, the causal relationship between elevated lactate and pain sensitization remains questionable, with ambiguous definitions of lactate-centered metabotypes compromising their reliability as clinical biomarkers. Furthermore, succinate receptor-targeting strategies face clinical translation challenges, primarily due to interspecific SUCNR1 distribution differences (Ren et al., 2022; Malatji et al., 2017; Natelson et al., 2017; Gerdle et al., 2020). Future studies should expand human chronic pain cohort sample analysis, integrate multidimensional metabolomics with targeted functional validation, elucidate the three metabolites’ clinical specificity and regulatory networks, and facilitate their clinical application as biomarkers and intervention targets.

## 6 Therapeutic strategies for resetting metabolite–neuro–immune coupling

**FIGURE 3:**
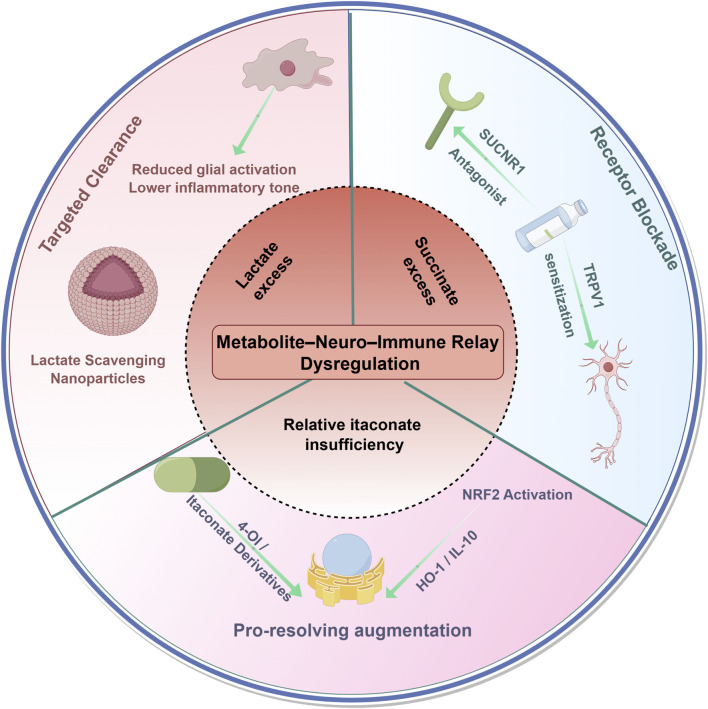
Therapeutic strategies targeting metabolite–neuro–immune relay dysregulation in chronic pain. This schematic outlines a metabolite-focused framework for correcting lactate excess, succinate excess and relative itaconate insufficiency that may sustain chronic pain. Targeted clearance aims to reduce extracellular lactate and acidosis within metabolically perturbed niches, exemplified by lactate-scavenging nanoparticles that can lower glial activation and inflammatory tone. Receptor blockade highlights inhibition of SUCNR1 on microglia and nociceptors to dampen pro-nociceptive Ca^2+^ signalling and limit TRPV1 sensitisation. Pro-resolving augmentation emphasises restoration of endogenous resolution pathways, including itaconate derivatives (for example, 4-OI) that may enhance NRF2-linked antioxidant and pro-resolving programmes (for example, HO-1 and IL-10).

Mechanistic work on lactate, succinate and itaconate has naturally prompted attempts to “re-wire” the metabolite–neuro–immune relay. Rather than adding another layer of broad anti-inflammatory drugs, these strategies intervene closer to the metabolic nodes that sustain neuroinflammation and neuronal sensitisation. Here we summarise four levels of intervention—enzyme and pathway modulation, metabolite clearance and receptor antagonism, next-generation delivery platforms and integrative treatment design—while avoiding repetition of mechanistic detail discussed above. Table 2 outlines a tiered landscape of strategies to reset pathological metabolite signalling in chronic pain. Figure 3 provides a schematic overview of therapeutic entry points targeting lactate excess, succinate excess and relative itaconate insufficiency.

**TABLE 2:** Therapeutic strategies targeting metabolite–neuro–immune dysregulation.

Strategy tier	Representative approach	Target	Expected effect
Pathway modulation	LDHA/glycolysis control	Lactate production	↓ pathological flux
Microenvironment remodelling	Lactate-scavenging materials	Extracellular lactate	↓ acidosis; ↓ glial tone
Receptor blockade	SUCNR1 antagonism	Succinate signalling	↓ pro-nociceptive Ca^2+^ signalling; ↓ TRPV1 sensitisation (preclinical)
Downstream buffering	ASIC/NLRP3 modulation	Lactate/succinate effects	↓ inflammatory amplification
Pro-resolving augmentation	4-OI/itaconate derivatives	Itaconate–NRF2 axis	↑ NRF2 target genes (e.g., HO-1) and pro-resolving cytokine tone (e.g., IL-10) (preclinical)
Precision delivery	pH/metabolite-responsive systems	Multi	Localised rebalancing

Unless specified, expected effects reflect preclinical proof-of-concept and mechanistic positioning within the metabolite–neuro–immune relay.

### 6.1 Metabolic enzyme inhibition and pathway reprogramming

Neuropathic pain is consistently associated with a glycolytic shift in spinal glia, and lactate dehydrogenase A (LDHA) has emerged as a tractable upstream node. In rodent models, intrathecal LDHA inhibitors such as FX11 or oxamate reduce spinal lactate accumulation, dampen microglial activation and reverse mechanical allodynia, consistent with a broader attenuation of lactate-driven inflammatory signalling (Cheng H. J. et al., 2024; Wen et al., 2023). These data support LDHA inhibition as a way to “cool down” spinal neuroinflammation by limiting pathological glycolytic flux rather than targeting individual cytokines.

Mitochondrial pathways offer a complementary lever. Pharmacological boosting of the IRG1–itaconate–NRF2 axis with cell-permeable derivatives (for example, 4-OI) reprograms macrophages and microglia toward resolution-biased states and reduces neuroinflammatory injury in several models (Liu et al., 2025; Zhao et al., 2025; Xia et al., 2023). However, because glycolytic and mitochondrial enzymes are ubiquitous, systemic modulation carries a clear risk of off-target toxicity in highly oxidative organs such as liver and skeletal muscle. Current efforts therefore prioritise regional delivery—via intrathecal catheters, implantable pumps or nanoparticle depots—to concentrate LDHA or succinate dehydrogenase modulators in pain-relevant tissue while sparing systemic metabolism.

### 6.2 Metabolite clearance, receptor antagonism and precision targeting

A more distal strategy is to remove excess metabolites or block their signalling “downstream”. Lactate scavenging is a prototypical example: nanozymes and injectable hydrogels equipped with lactate oxidase (often combined with catalase or other antioxidants) can consume extracellular lactate, neutralise local acidosis and attenuate inflammation in models of spinal cord or disc injury (Peng et al., 2024; Shen et al., 2022). Alkaline biomaterials such as magnesium oxide- or calcium carbonate-loaded hydrogels similarly buffer acidic lesions and indirectly dampen glial and immune activation, leaving upstream metabolic pathways largely intact while remodelling the microenvironment that sustains nociceptor sensitization (Park et al., 2024; Cai et al., 2025; Sergeeva et al., 2025).

Receptor-level interventions provide additional specificity. Antagonising SUCNR1 reduces cytokine release, T helper 17 cell expansion and hyperalgesia in arthritis models, in line with the succinate–SUCNR1–inflammasome axis described earlier (Saraiva et al., 2018; Zhang et al., 2024). Blockade of acid-sensing ion channels, which respond to lactate-associated acidosis, produces analgesic effects in several preclinical paradigms, although their contribution in human pain remains to be defined (Andreev et al., 2018). Modulating the lactate receptor HCAR1 is conceptually attractive but complicated by context-dependent pro- and anti-inflammatory actions and therefore remains exploratory.

Spatial metabolomics adds a precision layer to these approaches. Techniques such as matrix-assisted laser desorption/ionisation mass spectrometry imaging can reveal focal hotspots of glycolysis, lactate or succinate that co-localise with glial scars, specific dorsal horn laminae or DRG subpopulations. In principle, such maps could guide site-specific deployment of lactate-scavenging systems, pH-buffering materials or SUCNR1 antagonists, turning diffuse metabolic interventions into anatomically targeted therapies.

### 6.3 Emerging technologies: nanodelivery and synthetic biology

Next-generation delivery systems are being engineered to match the spatial and temporal complexity of metabolic signalling. Stimuli-responsive nanocarriers with pH- or metabolite-sensitive coatings can circulate systemically but remain inert in healthy tissue, releasing anti-inflammatory or neuroprotective drugs only within acidic, lactate-rich microenvironments. Catalytic nanozymes extend this concept by embedding enzyme-like activity directly into nanomaterials; constructs with lactate oxidase-mimetic properties, for example, can deplete lactate *in situ* and reshape local immune tone (Yang et al., 2024; Cheng Q. et al., 2024). Similar platforms could, in principle, be tailored to modulate succinate or other pain-linked metabolites.

In parallel, synthetic biology offers a complementary, cell-based route. Probiotic strains have been programmed to secrete interleukin-10 or to activate therapeutic circuits in response to endogenous inflammatory cues in non-neurological diseases (Hong et al., 2021; Weibel et al., 2024). Extrapolated to pain, “smart” microbes could be designed to sense elevated lactate or succinate in peripheral tissues and, only under those conditions, release anti-inflammatory cytokines, metabolic modulators or small-molecule analgesics. Although still at the proof-of-concept stage for nociceptive pathways, these metabolite-responsive, feedback-controlled systems illustrate how immunometabolic modulation could be made both highly local and dynamically adaptive.

### 6.4 Strategic considerations and mechanistic implications

The interventions outlined above operate at different tiers of the metabolic network—from pathway reprogramming (LDHA or mitochondrial enzymes) to microdomain-level modulation (scavengers, buffers and receptor antagonists) and advanced delivery systems. Broad-spectrum approaches are powerful but demand careful localisation to avoid systemic toxicity, whereas downstream strategies naturally lend themselves to regional application. Multimodal spatial metabolomics, especially when combined with spatial immunophenotyping, can help identify the most disturbed microenvironments and match them with appropriate tools.

A staged, hybrid treatment paradigm is therefore attractive. An initial “reset” phase would normalise pathological metabolic niches—for example, using nanozymes or buffering hydrogels to reduce acidosis and excess lactate—followed by a maintenance phase in which enzyme inhibitors or receptor antagonists stabilise the new set point (Li et al., 2023). Metabolomic biomarkers could be used both to stratify patients according to dominant metabolic derangements (for instance, lactate-centred versus succinate-centred) and to monitor response over time. The rapid progress of stimuli-responsive nanomedicines in other inflammatory conditions suggests that a metabolically informed, spatially resolved framework for chronic pain therapy is a realistic, rather than purely speculative, goal.

In summary, regarding the clinical translation of metabolic regulation strategies, current research still has key controversies and bottlenecks in the pathways, targets, and delivery levels: in the intervention of pathways such as LDHA and the IRG1-itaconate-NRF2 axis, the balance between systemic toxicity and the effectiveness of regional delivery remains unclear; the humanized validation of receptor-level strategies such as SUCNR1 antagonism is insufficient, and the transformation barriers related to species differences have not yet been broken; the delivery systems mediated by nanocarriers and synthetic biology are mostly in the proof-of-concept stage, with limited clinical translation data (Li et al., 2023; Cheng H. J. et al., 2024; Ren et al., 2022; Yang et al., 2024). Future studies should focus on optimizing the safety of delivery systems, validating humanized targets, and evaluating combined multi-strategy regimens to promote the transformation of metabolic regulation-based analgesic therapies from preclinical to clinical applications.

## 7 Conclusion

Chronic pain has traditionally been described through the lenses of neuronal hyperexcitability, synaptic plasticity and neuroinflammation (Ji et al., 2016; Kuner and Flor, 2016). The evidence synthesised in this Review adds a complementary layer: persistent pain is sustained, in part, by a metabolite–neuro–immune relay in which lactate and succinate function as context-dependent danger signals, whereas itaconate provides an endogenous brake. Upstream metabolic reprogramming of astrocytes, microglia and peripheral immune cells generates characteristic patterns of these metabolites; glial cells decode these cues via HIF-1α, NF-κB, NLRP3 and NRF2 pathways; and the resulting cytokines, growth factors and physicochemical changes converge on nociceptors and spinal circuits to maintain central sensitisation. In this framework, chronic pain is not simply a property of overactive neurons, but a state in which neural circuits are continuously retuned by their metabolic microenvironment.

Human data, although still more limited than preclinical work, reinforce this view. Metabolomic analyses in fibromyalgia and osteoarthritis have consistently revealed alterations in energy-related intermediates such as lactate and succinate across plasma, muscle, and synovial fluid, correlating with pain intensity and fatigue (Piras et al., 2022; Gerdle et al., 2024; Farah et al., 2022; Favretti et al., 2023). These metabolic pain phenotypes converge on pathways highlighted by experimental studies—glycolytic shifts, TCA cycle imbalance and inflammasome activation—yet differ between syndromes and even between subgroups within a single diagnosis. Such heterogeneity argues against a one-size-fits-all biomarker, but strongly supports the use of metabolomics to define endotypes, select patients for mechanism-based trials and monitor metabolic responses to treatment.

The same relay model also helps to organise emerging therapeutic strategies. Enzyme-directed approaches, such as inhibition of LDHA or pharmacological reinforcement of the IRG1–itaconate–NRF2 axis, attenuate glial activation and nociceptive hypersensitivity in preclinical models by acting upstream of cytokine production (Cheng H. J. et al., 2024; Jha et al., 2015; Mills et al., 2018). Metabolite clearance systems and receptor antagonists—lactate-scavenging nanozymes, pH-buffering biomaterials, SUCNR1 blockers, inhibitors of acid-sensing ion channels or the NLRP3 inflammasome—offer more spatially confined ways to interrupt pathological signalling (Mohsin et al., 2025; Zhao et al., 2021; Yuan et al., 2022; Chu et al., 2024; Silva Santos Ribeiro et al., 2023). Nanomedicine and synthetic biology extend these ideas further, enabling stimulus-responsive drug delivery and engineered microbes that release anti-inflammatory mediators only in metabolically perturbed niches. Although most of these approaches remain at the proof-of-concept stage, they demonstrate how a mechanistic understanding of metabolite signalling can be translated into concrete, testable interventions.

To fully decipher the metabolite–neuro–immune relay in chronic pain and translate mechanistic insights into clinical practice, future research must integrate advanced technologies with targeted scientific questions. Spatial metabolomics, when combined with spatial transcriptomics and functional imaging, addresses the core gap of unclear causal relationships between metabolite-enriched microdomains and neuroimmune activation—enabling precise mapping of lactate/succinate hotspots in human dorsal root ganglia and spinal dorsal horn, and clarifying whether these metabolic niches drive or merely accompany glial activation and neuronal sensitization. Single-cell multi-omics resolves the ambiguity of cell-type-specific metabolic regulation, dissecting how astrocytes, microglia, and sensory neurons uniquely respond to lactate, succinate, and itaconate, and identifying cell-subtype-specific therapeutic nodes to overcome pathway redundancy. Genetically encoded sensors and *in vivo* dynamic imaging tackle the technical bottleneck of real-time metabolite tracking, quantifying how lactate/succinate/itaconate concentrations fluctuate across pain phases and correlating these dynamics with neuronal excitability, thus unraveling the temporal coupling of metabolic signals to neuroinflammation. Complemented by human-induced pluripotent stem cell-derived spinal organoids and assembloids, these approaches bridge species differences inherent in rodent models, validating metabolite targets (e.g., SUCNR1, IRG1) and intervention strategies in human-relevant systems. Collectively, this integrated framework will clarify the molecular logic of metabolic network imbalance, establish metabolite-based patient stratification, and accelerate the development of precision analgesics that reset the pathological relay while preserving physiological homeostasis. Taken together, current data support a pragmatic, testable proposition: chronic pain is sustained by metabolically encoded signals that couple tissue stress to neuroimmune plasticity. Lactate, succinate and itaconate exemplify this logic, but are unlikely to be unique. The central challenge now is to determine when such metabolites act as drivers, modifiers or mere correlates of disease, and how the pathological relay they participate in can be safely reset in patients. Achieving this will require close integration of mechanistic studies, spatial–temporal mapping and biomarker-guided clinical trials. If successful, it should enable a shift from empirical analgesia toward therapies that are designed, timed and delivered according to each patient’s metabolic landscape—realising, in practice, the metabolite–neuro–immune relay model outlined in this Review.

## References

[B1] Agarwal A. Wu P. H. Hughes E. G. Fukaya M. Tischfield M. A. Langseth A. J. (2017). Transient opening of the mitochondrial permeability transition pore induces microdomain calcium transients in astrocyte processes. Neuron 93 (3), 587–605. 10.1016/j.neuron.2016.12.034 28132831 PMC5308886

[B2] Ahmadpour N. Kantroo M. Stobart J. L. (2021). Extracellular calcium influx pathways in astrocyte calcium microdomain physiology. Biomolecules 11 (10), 1467. 10.3390/biom11101467 34680100 PMC8533159

[B3] Andreev Y. A. Osmakov D. I. Koshelev S. G. Maleeva E. E. Logashina Y. A. Palikov V. A. (2018). Analgesic activity of acid-sensing ion channel 3 (ASIС3) inhibitors: sea anemones peptides Ugr9-1 and APETx2 versus low molecular weight compounds. Mar. Drugs 16 (12), 500. 10.3390/md16120500 PMC6316600 30545037

[B4] Atta A. A. Ibrahim W. W. Mohamed A. F. Abdelkader N. F. (2023). Microglia polarization in nociplastic pain: mechanisms and perspectives. Inflammopharmacology 31 (3), 1053–1067. 10.1007/s10787-023-01216-x 37069462 PMC10229465

[B5] Behl T. Makkar R. Anwer M. K. Hassani R. Khuwaja G. Khalid A. (2023). Mitochondrial dysfunction: a cellular and molecular hub in pathology of metabolic diseases and infection. J. Clin. Med. 12 (8), 2882. 10.3390/jcm12082882 37109219 PMC10141031

[B6] Belo T. C. A. Santos G. X. da Silva B. E. G. Rocha B. L. G. Abdala D. W. Freire L. A. M. (2023). IL-10/β-Endorphin-Mediated neuroimmune modulation on microglia during antinociception. Brain Sci. 13 (5), 789. 10.3390/brainsci13050789 37239261 PMC10216064

[B7] Bian Z. Guo T. Jiang S. Chen L. Liu J. Zheng G. (2021). High-throughput functional characterization of visceral afferents by optical recordings from thoracolumbar and Lumbosacral dorsal root ganglia. Front. Neurosci. 15, 657361. 10.3389/fnins.2021.657361 33776645 PMC7991386

[B8] Boakye P. A. Tang S. J. Smith P. A. (2021). Mediators of neuropathic pain; focus on spinal microglia, CSF-1, BDNF, CCL21, TNF-α, wnt ligands, and interleukin 1β. Front. Pain Res. (Lausanne) 2, 698157. 10.3389/fpain.2021.698157 35295524 PMC8915739

[B9] Cai L. Huang J. Huang D. Lv H. Wang D. Wang H. (2023). Deficiency of immune-responsive gene 1 exacerbates interleukin-1beta-elicited the inflammatory response of chondrocytes via enhancing the activation of NLRP3 inflammasome. Int. Immunopharmacol. 114, 109456. 10.1016/j.intimp.2022.109456 36442283

[B10] Cai Y. Chen Y. Li H. Wang Y. Zhang G. Liang J. (2025). Fabrication of GDNF-Gel/HA-Mg nerve conduit and its role in repairing peripheral nerve defects. Mater. Today Bio 32, 101764. 10.1016/j.mtbio.2025.101764 40290886 PMC12022700

[B11] Chang J. Li Z. Yuan H. Wang X. Xu J. Yang P. (2024). Protective role of aconitate decarboxylase 1 in neuroinflammation-induced dysfunctions of the paraventricular thalamus and sleepiness. Commun. Biol. 7 (1), 1484. 10.1038/s42003-024-07215-0 39523388 PMC11551151

[B12] Chen C. Zhang J. Sun L. Zhang Y. Gan W. B. Tang P. (2019). Long-term imaging of dorsal root ganglia in awake behaving mice. Nat. Commun. 10 (1), 3087. 10.1038/s41467-019-11158-0 31300648 PMC6625980

[B13] Chen R. Hu J. Zhang Y. Liu Y. Zhu J. Pan Z. (2024). Total glucosides of paeony ameliorates chemotherapy-induced neuropathic pain by suppressing microglia pyroptosis through the inhibition of KAT2A-mediated p38 pathway activation and succinylation. Sci. Rep. 14 (1), 31875. 10.1038/s41598-024-83207-8 39738348 PMC11686281

[B14] Chen L. Lin Y. Zhu X. Zhuo S. Li Z. Guo C. (2025). MCT1-mediated lactate shuttle to mitochondria governs macrophage polarization and modulates glucose homeostasis by affecting β cells. Adv. Sci. (Weinh) 12 (38), e14760. 10.1002/advs.202414760 40660708 PMC12520533

[B15] Cheng H. Zhang L. Xia F. Jin L. Liu S. Ren H. (2020). Astrocytic NDRG2 is critical in the maintenance of neuropathic pain. Brain Behav. Immun. 89, 300–313. 10.1016/j.bbi.2020.07.009 32688030

[B16] Cheng J. Zhang R. Xu Z. Ke Y. Sun R. Yang H. (2021). Early glycolytic reprogramming controls microglial inflammatory activation. J. Neuroinflammation 18 (1), 129. 10.1186/s12974-021-02187-y 34107997 PMC8191212

[B17] Cheng H. J. Chen N. F. Chen W. F. Wu Z. S. Sun Y. Y. Teng W. N. (2024). Intrathecal lactate dehydrogenase A inhibitors FX11 and oxamate alleviate chronic constriction injury-induced nociceptive sensitization through neuroinflammation and angiogenesis. J. Headache Pain 25 (1), 207. 10.1186/s10194-024-01916-x 39587478 PMC11590346

[B18] Cheng Q. Shi X. L. Li Q. L. Wang L. Wang Z. (2024). Current advances on nanomaterials interfering with lactate metabolism for tumor therapy. Adv. Sci. (Weinh) 11 (3), e2305662. 10.1002/advs.202305662 37941489 PMC10797484

[B19] Chu J. Yang J. Zhou Y. Chen J. Chen K. H. Zhang C. (2023). ATP-releasing SWELL1 channel in spinal microglia contributes to neuropathic pain. Sci. Adv. 9 (13), eade9931. 10.1126/sciadv.ade9931 36989353 PMC10058245

[B20] Chu Y. Wu Y. Jia S. Xu K. Liu J. Mai L. (2023). Single-nucleus transcriptome analysis reveals transcriptional profiles of circadian clock and pain related genes in human and mouse trigeminal ganglion. Front. Neurosci. 17, 1176654. 10.3389/fnins.2023.1176654 37250405 PMC10210144

[B21] Chu D. Zhao M. Rong S. Jhe W. Cai X. Xiao Y. (2024). Dual-atom nanozyme eye drops attenuate inflammation and break the vicious cycle in dry eye disease. Nanomicro Lett. 16 (1), 120. 10.1007/s40820-024-01322-7 38372846 PMC10876514

[B22] Clos-Garcia M. Andrés-Marin N. Fernández-Eulate G. Abecia L. Lavín J. L. van Liempd S. (2019). Gut microbiome and serum metabolome analyses identify molecular biomarkers and altered glutamate metabolism in fibromyalgia. EBioMedicine 46, 499–511. 10.1016/j.ebiom.2019.07.031 31327695 PMC6710987

[B23] Cooper M. L. Pasini S. Lambert W. S. D'Alessandro K. B. Yao V. Risner M. L. (2020). Redistribution of metabolic resources through astrocyte networks mitigates neurodegenerative stress. Proc. Natl. Acad. Sci. U. S. A. 117 (31), 18810–18821. 10.1073/pnas.2009425117 32690710 PMC7414143

[B24] Darvish K. M. Tabandeh M. R. Haschemi A. Kheirollah A. Shahriari A. (2022). Dimethyl itaconate reprograms neurotoxic to neuroprotective primary astrocytes through the regulation of NLRP3 inflammasome and NRF2/HO-1 pathways. Mol. Cell Neurosci. 122, 103758. 10.1016/j.mcn.2022.103758 35868484

[B25] de Castro Abrantes H. Briquet M. Schmuziger C. Restivo L. Puyal J. Rosenberg N. (2019). The lactate receptor HCAR1 modulates neuronal network activity through the activation of G(α) and G(βγ) subunits. J. Neurosci. 39 (23), 4422–4433. 10.1523/jneurosci.2092-18.2019 30926749 PMC6554634

[B26] de Macedo F. H. P. Aires R. D. Fonseca E. G. Ferreira R. C. M. Machado D. P. D. Chen L. (2019). TNF-α mediated upregulation of Na(V)1.7 currents in rat dorsal root ganglion neurons is independent of CRMP2 SUMOylation. Mol. Brain 12 (1), 117. 10.1186/s13041-019-0538-0 31888677 PMC6937926

[B27] Echeverria-Villalobos M. Tortorici V. Brito B. E. Ryskamp D. Uribe A. Weaver T. (2023). The role of neuroinflammation in the transition of acute to chronic pain and the opioid-induced hyperalgesia and tolerance. Front. Pharmacol. 14, 1297931. 10.3389/fphar.2023.1297931 38161698 PMC10755684

[B28] Farah H. Wijesinghe S. N. Nicholson T. Alnajjar F. Certo M. Alghamdi A. (2022). Differential metabotypes in synovial fibroblasts and synovial fluid in hip osteoarthritis patients support inflammatory responses. Int. J. Mol. Sci. 23 (6), 3266. 10.3390/ijms23063266 35328687 PMC8950319

[B29] Favret S. Binet F. Lapalme E. Leboeuf D. Carbadillo J. Rubic T. (2013). Deficiency in the metabolite receptor SUCNR1 (GPR91) leads to outer retinal lesions. Aging (Albany NY) 5 (6), 427–444. 10.18632/aging.100563 23833031 PMC3832265

[B30] Favretti M. Iannuccelli C. Di Franco M. (2023). Pain biomarkers in fibromyalgia syndrome: current understanding and future directions. Int. J. Mol. Sci. 24 (13), 10443. 10.3390/ijms241310443 37445618 PMC10341963

[B31] Fineschi S. Klar J. Gustafsson K. A. Jonsson K. Karlsson B. Dahl N. (2022). Inflammation and interferon signatures in peripheral B-Lymphocytes and sera of individuals with fibromyalgia. Front. Immunol. 13, 874490. 10.3389/fimmu.2022.874490 35693781 PMC9177944

[B32] Gerdle B. Söderberg K. Salvador Puigvert L. Rosendal L. Larsson B. (2010). Increased interstitial concentrations of pyruvate and lactate in the trapezius muscle of patients with fibromyalgia: a microdialysis study. J. Rehabil. Med. 42 (7), 679–687. 10.2340/16501977-0581 20603699

[B33] Gerdle B. Ghafouri B. Lund E. Bengtsson A. Lundberg P. Ettinger-Veenstra H. V. (2020). Evidence of mitochondrial dysfunction in fibromyalgia: deviating muscle energy metabolism detected using microdialysis and magnetic resonance. J. Clin. Med. 9 (11), 3527. 10.3390/jcm9113527 33142767 PMC7693920

[B34] Gerdle B. Dahlqvist Leinhard O. Lund E. Lundberg P. Forsgren M. F. Ghafouri B. (2024). Pain and the biochemistry of fibromyalgia: patterns of peripheral cytokines and chemokines contribute to the differentiation between fibromyalgia and controls and are associated with pain, fat infiltration and content. Front. Pain Res. (Lausanne) 5, 1288024. 10.3389/fpain.2024.1288024 38304854 PMC10830731

[B35] Gilissen J. Jouret F. Pirotte B. Hanson J. (2016). Insight into SUCNR1 (GPR91) structure and function. Pharmacol. Ther. 159, 56–65. 10.1016/j.pharmthera.2016.01.008 26808164

[B36] Guo Y. Xu F. Thomas S. C. Zhang Y. Paul B. Sakilam S. (2022). Targeting the succinate receptor effectively inhibits periodontitis. Cell Rep. 40 (12), 111389. 10.1016/j.celrep.2022.111389 36130514 PMC9533417

[B37] He J. H. Yu L. Wang Z. Y. Wang Q. Cao J. L. Gu L. B. (2019). Inhibition of monocarboxylate transporter 1 in spinal cord horn significantly reverses chronic inflammatory pain. J. Pain Res. 12, 2981–2990. 10.2147/jpr.S219359 31807055 PMC6842320

[B38] He L. Yin R. Hang W. Han J. Chen J. Wen B. (2024). Oxygen glucose deprivation-induced lactylation of H3K9 contributes to M1 polarization and inflammation of microglia through TNF pathway. Biomedicines 12 (10), 2371. 10.3390/biomedicines12102371 39457683 PMC11504212

[B39] Hong N. Ku S. Yuk K. Johnston T. V. Ji G. E. Park M. S. (2021). Production of biologically active human interleukin-10 by Bifidobacterium bifidum BGN4. Microb. Cell Fact. 20 (1), 16. 10.1186/s12934-020-01505-y 33468130 PMC7814708

[B40] Hooftman A. Angiari S. Hester S. Corcoran S. E. Runtsch M. C. Ling C. (2020). The immunomodulatory metabolite itaconate modifies NLRP3 and inhibits inflammasome activation. Cell Metab. 32 (3), 468–478.e467. 10.1016/j.cmet.2020.07.016 32791101 PMC7422798

[B41] Hu Y. Zou H. Zhong Z. Li Q. Zeng Q. Ouyang Q. (2024). The role of astrocyte-neuron lactate shuttle in neuropathic orofacial pain. J. Oral Rehabil. 51 (12), 2513–2528. 10.1111/joor.13847 39209792

[B42] Hua T. Kong E. Zhang H. Lu J. Huang K. Ding R. (2024). PRMT6 deficiency or inhibition alleviates neuropathic pain by decreasing glycolysis and inflammation in microglia. Brain Behav. Immun. 118, 101–114. 10.1016/j.bbi.2024.02.027 38402915

[B43] Huang H. Li G. He Y. Chen J. Yan J. Zhang Q. (2024). Cellular succinate metabolism and signaling in inflammation: implications for therapeutic intervention. Front. Immunol. 15, 1404441. 10.3389/fimmu.2024.1404441 38933270 PMC11200920

[B44] Jha M. K. Song G. J. Lee M. G. Jeoung N. H. Go Y. Harris R. A. (2015). Metabolic connection of inflammatory pain: pivotal role of a pyruvate dehydrogenase kinase-pyruvate dehydrogenase-lactic acid axis. J. Neurosci. 35 (42), 14353–14369. 10.1523/jneurosci.1910-15.2015 26490872 PMC6605420

[B45] Ji R. R. Chamessian A. Zhang Y. Q. (2016). Pain regulation by non-neuronal cells and inflammation. Science 354 (6312), 572–577. 10.1126/science.aaf8924 27811267 PMC5488328

[B46] Kambe Y. Youkai M. Hashiguchi K. Sameshima Y. Takasaki I. Miyata A. (2022). Spinal astrocyte-neuron lactate shuttle contributes to the pituitary adenylate cyclase-activating Polypeptide/PAC1 receptor-induced nociceptive behaviors in mice. Biomolecules 12 (12), 1859. 10.3390/biom12121859 36551287 PMC9775268

[B47] Keiran N. Ceperuelo-Mallafré V. Calvo E. Hernández-Alvarez M. I. Ejarque M. Núñez-Roa C. (2019). SUCNR1 controls an anti-inflammatory program in macrophages to regulate the metabolic response to obesity. Nat. Immunol. 20 (5), 581–592. 10.1038/s41590-019-0372-7 30962591

[B48] Kim H. K. Lee S. Y. Koike N. Kim E. Wirianto M. Burish M. J. (2020). Circadian regulation of chemotherapy-induced peripheral neuropathic pain and the underlying transcriptomic landscape. Sci. Rep. 10 (1), 13844. 10.1038/s41598-020-70757-w 32796949 PMC7427990

[B49] Kiritoshi T. Yakhnitsa V. Singh S. Wilson T. D. Chaudhry S. Neugebauer B. (2024). Cells and circuits for amygdala neuroplasticity in the transition to chronic pain. Cell Rep. 43 (9), 114669. 10.1016/j.celrep.2024.114669 39178115 PMC11473139

[B50] Kong L. Wang Z. Liang X. Wang Y. Gao L. Ma C. (2019). Monocarboxylate transporter 1 promotes classical microglial activation and pro-inflammatory effect via 6-phosphofructo-2-kinase/fructose-2, 6-biphosphatase 3. J. Neuroinflammation 16 (1), 240. 10.1186/s12974-019-1648-4 31779643 PMC6883695

[B51] Kong E. Li Y. Deng M. Hua T. Yang M. Li J. (2022). Glycometabolism reprogramming of glial cells in central nervous system: novel target for neuropathic pain. Front. Immunol. 13, 861290. 10.3389/fimmu.2022.861290 35669777 PMC9163495

[B52] Kong E. Li Y. Ma P. Zhang Y. Ding R. Hua T. (2023). Lyn-mediated glycolysis enhancement of microglia contributes to neuropathic pain through facilitating IRF5 nuclear translocation in spinal dorsal horn. J. Cell Mol. Med. 27 (12), 1664–1681. 10.1111/jcmm.17759 37132040 PMC10273059

[B53] Kong X. Lyu W. Lin X. Lin C. Feng H. Xu L. (2024). Itaconate alleviates anesthesia/surgery-induced cognitive impairment by activating a Nrf2-dependent anti-neuroinflammation and neurogenesis via gut-brain axis. J. Neuroinflammation 21 (1), 104. 10.1186/s12974-024-03103-w 38649932 PMC11034021

[B54] Kong Z. Jiang J. Deng M. Deng M. Wu H. (2025). Improving epilepsy management by targeting P2 × 7 receptor with ROS/electric responsive nanomicelles. J. Nanobiotechnology 23 (1), 332. 10.1186/s12951-025-03386-y 40325469 PMC12054225

[B55] Kuner R. Flor H. (2016). Structural plasticity and reorganisation in chronic pain. Nat. Rev. Neurosci. 18 (1), 20–30. 10.1038/nrn.2016.162 27974843

[B56] Kuo P. C. Weng W. T. Scofield B. A. Furnas D. Paraiso H. C. Yu I. C. (2021). Immunoresponsive gene 1 modulates the severity of brain injury in cerebral ischaemia. Brain Commun. 3 (3), fcab187. 10.1093/braincomms/fcab187 34557667 PMC8453405

[B57] Lampropoulou V. Sergushichev A. Bambouskova M. Nair S. Vincent E. E. Loginicheva E. (2016). Itaconate links inhibition of succinate dehydrogenase with macrophage metabolic remodeling and regulation of inflammation. Cell Metab. 24 (1), 158–166. 10.1016/j.cmet.2016.06.004 27374498 PMC5108454

[B58] Lauterbach M. A. Hanke J. E. Serefidou M. Mangan M. S. J. Kolbe C. C. Hess T. (2019). Toll-like receptor signaling rewires macrophage metabolism and promotes histone acetylation via ATP-citrate lyase. Immunity 51 (6), 997–1011.e1017. 10.1016/j.immuni.2019.11.009 31851905

[B59] Li J. Gong C. Chen X. Guo H. Tai Z. Ding N. (2023). Biomimetic liposomal nanozymes improve breast cancer chemotherapy with enhanced penetration and alleviated hypoxia. J. Nanobiotechnology 21 (1), 123. 10.1186/s12951-023-01874-7 37038165 PMC10084658

[B60] Li Y. Jin J. Kang X. Feng Z. (2024a). Identifying and evaluating biological markers of postherpetic neuralgia: a comprehensive review. Pain Ther. 13 (5), 1095–1117. 10.1007/s40122-024-00640-3 39126594 PMC11393369

[B61] Li Y. Kong E. Ding R. Chu R. Lu J. Deng M. (2024b). Hyperglycemia-induced Sirt3 downregulation increases microglial aerobic glycolysis and inflammation in diabetic neuropathic pain pathogenesis. CNS Neurosci. Ther. 30 (8), e14913. 10.1111/cns.14913 39123294 PMC11315676

[B62] Lin J. Ren J. Zhu B. Dai Y. Gao D. S. Xia S. (2022). Dimethyl itaconate attenuates CFA-induced inflammatory pain via the NLRP3/IL-1β signaling pathway. Front. Pharmacol. 13, 938979. 10.3389/fphar.2022.938979 35935847 PMC9353300

[B63] Littlewood-Evans A. Sarret S. Apfel V. Loesle P. Dawson J. Zhang J. (2016). GPR91 senses extracellular succinate released from inflammatory macrophages and exacerbates rheumatoid arthritis. J. Exp. Med. 213 (9), 1655–1662. 10.1084/jem.20160061 27481132 PMC4995082

[B64] Liu H. Feng Y. Xu M. Yang J. Wang Z. Di G. (2018). Four-octyl itaconate activates Keap1-Nrf2 signaling to protect neuronal cells from hydrogen peroxide. Cell Commun. Signal 16 (1), 81. 10.1186/s12964-018-0294-2 30442144 PMC6238317

[B65] Liu S. Yang S. Zhu X. Li X. Zhang X. Zhou X. (2023). Spinal apolipoprotein E is involved in inflammatory pain via regulating lipid metabolism and glial activation in the spinal dorsal horn. Biol. Direct 18 (1), 85. 10.1186/s13062-023-00444-z 38071369 PMC10710718

[B66] Liu N. Jiang Y. Xiu Y. Tortelote G. G. Xia W. Wang Y. (2025). Itaconate restrains acute proinflammatory activation of microglia after traumatic brain injury in mice. Sci. Transl. Med. 17 (789), eadn2635. 10.1126/scitranslmed.adn2635 40073156

[B67] López-Moyado I. F. Ko M. Hogan P. G. Rao A. (2024). TET enzymes in the immune system: from DNA demethylation to immunotherapy, inflammation, and. Cancer. Annu. Rev. Immunol. 42 (1), 455–488. 10.1146/annurev-immunol-080223-044610 38360546

[B68] Luo L. Zhuang X. Fu L. Dong Z. Yi S. Wang K. (2024). The role of the interplay between macrophage glycolytic reprogramming and NLRP3 inflammasome activation in acute lung injury/acute respiratory distress syndrome. Clin. Transl. Med. 14 (12), e70098. 10.1002/ctm2.70098 39623879 PMC11612265

[B69] Ma K. Ding X. Song Q. Han Z. Yao H. Ding J. (2020). Lactate enhances Arc/arg3.1 expression through hydroxycarboxylic acid receptor 1-β-arrestin2 pathway in astrocytes. Neuropharmacology 171, 108084. 10.1016/j.neuropharm.2020.108084 32294462

[B70] Ma X. Qi Q. Wang W. Huang M. Wang H. Luo L. (2024). Astrocytic pyruvate dehydrogenase kinase-lactic acid axis involvement in glia-neuron crosstalk contributes to morphine-induced hyperalgesia in mice. Fundam. Res. 4 (4), 820–828. 10.1016/j.fmre.2023.02.013 39161415 PMC11331729

[B71] Macchi C. Giachi A. Fichtner I. Pedretti S. Sarzi-Puttini P. Mitro N. (2024). Mitochondrial function in patients affected with fibromyalgia syndrome is impaired and correlates with disease severity. Sci. Rep. 14 (1), 30247. 10.1038/s41598-024-81298-x 39632893 PMC11618515

[B72] Malatji B. G. Meyer H. Mason S. Engelke U. F. H. Wevers R. A. van Reenen M. (2017). A diagnostic biomarker profile for fibromyalgia syndrome based on an NMR metabolomics study of selected patients and controls. BMC Neurol. 17 (1), 88. 10.1186/s12883-017-0863-9 28490352 PMC5426044

[B73] Marty-Lombardi S. Lu S. Ambroziak W. Schrenk-Siemens K. Wang J. DePaoli-Roach A. A. (2024). Neuron-astrocyte metabolic coupling facilitates spinal plasticity and maintenance of inflammatory pain. Nat. Metab. 6 (3), 494–513. 10.1038/s42255-024-01001-2 38443593 PMC10963271

[B74] McKinnon J. C. Balez R. Young R. S. E. Brown M. L. Lum J. S. Robinson L. (2024). MALDI-2-Enabled oversampling for the mass spectrometry imaging of metabolites at single-cell resolution. J. Am. Soc. Mass Spectrom. 35 (11), 2729–2742. 10.1021/jasms.4c00241 39137242

[B75] Miao J. Chen L. Pan X. Li L. Zhao B. Lan J. (2023). Microglial metabolic reprogramming: emerging insights and therapeutic strategies in neurodegenerative diseases. Cell Mol. Neurobiol. 43 (7), 3191–3210. 10.1007/s10571-023-01376-y 37341833 PMC11410021

[B76] Mills E. L. Ryan D. G. Prag H. A. Dikovskaya D. Menon D. Zaslona Z. (2018). Itaconate is an anti-inflammatory metabolite that activates Nrf2 via alkylation of KEAP1. Nature 556 (7699), 113–117. 10.1038/nature25986 29590092 PMC6047741

[B77] Miyamoto K. Ishikura K. I. Kume K. Ohsawa M. (2019). Astrocyte-neuron lactate shuttle sensitizes nociceptive transmission in the spinal cord. Glia 67 (1), 27–36. 10.1002/glia.23474 30430652

[B78] Mohsin M. Shams F. Li H. Alam A. Xia C. Fan L. (2025). Nanozymes in neuropathic pain: strategies bridging oxidative stress, mitochondrial repair, and neuroimmune modulation for targeted therapy. J. Neuroinflammation 22 (1), 156. 10.1186/s12974-025-03456-w 40506712 PMC12164103

[B79] Molnár T. Dobolyi A. Nyitrai G. Barabás P. Héja L. Emri Z. (2011). Calcium signals in the nucleus accumbens: activation of astrocytes by ATP and succinate. BMC Neurosci. 12, 96. 10.1186/1471-2202-12-96 21967230 PMC3199278

[B80] Murphy-Royal C. Johnston A. D. Boyce A. K. J. Diaz-Castro B. Institoris A. Peringod G. (2020). Stress gates an astrocytic energy reservoir to impair synaptic plasticity. Nat. Commun. 11 (1), 2014. 10.1038/s41467-020-15778-9 32332733 PMC7181611

[B81] Natelson B. H. Vu D. Mao X. Weiduschat N. Togo F. Lange G. (2015). Effect of milnacipran treatment on ventricular lactate in fibromyalgia: a randomized, double-blind, placebo-controlled trial. J. Pain 16 (11), 1211–1219. 10.1016/j.jpain.2015.08.004 26335989 PMC4630071

[B82] Natelson B. H. Vu D. Coplan J. D. Mao X. Blate M. Kang G. (2017). Elevations of ventricular lactate levels occur in both chronic fatigue syndrome and fibromyalgia. Fatigue 5 (1), 15–20. 10.1080/21641846.2017.1280114 29308330 PMC5754037

[B83] Ni Z. Kuang L. Chen H. Xie Y. Zhang B. Ouyang J. (2019). The exosome-like vesicles from osteoarthritic chondrocyte enhanced mature IL-1β production of macrophages and aggravated synovitis in osteoarthritis. Cell Death Dis. 10 (7), 522. 10.1038/s41419-019-1739-2 31285423 PMC6614358

[B84] Ni L. Xiao J. Zhang D. Shao Z. Huang C. Wang S. (2022). Immune-responsive gene 1/itaconate activates nuclear factor erythroid 2-related factor 2 in microglia to protect against spinal cord injury in mice. Cell Death Dis. 13 (2), 140. 10.1038/s41419-022-04592-4 35145070 PMC8831631

[B85] Nunns G. R. Vigneshwar N. Kelher M. R. Stettler G. R. Gera L. Reisz J. A. (2022). Succinate activation of SUCNR1 predisposes severely injured patients to neutrophil-mediated ARDS. Ann. Surg. 276 (6), e944–e954. 10.1097/sla.0000000000004644 33214479 PMC8128932

[B86] Ohm M. Hosseini S. Lonnemann N. He W. More T. Goldmann O. (2024). The potential therapeutic role of itaconate and mesaconate on the detrimental effects of LPS-induced neuroinflammation in the brain. J. Neuroinflammation 21 (1), 207. 10.1186/s12974-024-03188-3 39164713 PMC11337794

[B87] Pan X. Ye F. Ning P. Yu Y. Zhang Z. Wang J. (2025). Structures of G-protein coupled receptor HCAR1 in complex with Gi1 protein reveal the mechanistic basis for ligand recognition and agonist selectivity. PLoS Biol. 23 (4), e3003126. 10.1371/journal.pbio.3003126 40233099 PMC12040280

[B88] Park S. Y. Jung J. H. Kim D. S. Lee J. K. Song B. G. Shin H. E. (2024). Therapeutic potential of luteolin-loaded poly(lactic-co-glycolic acid)/modified magnesium hydroxide microsphere in functional thermosensitive hydrogel for treating neuropathic pain. J. Tissue Eng. 15, 20417314231226105. 10.1177/20417314231226105 38333057 PMC10851718

[B89] Peng Y. Chen X. Zhang Q. Liu S. Wu W. Li K. (2024). Enzymatically bioactive nucleus pulposus matrix hydrogel microspheres for exogenous stem cells therapy and endogenous repair strategy to achieve disc regeneration. Adv. Sci. (Weinh) 11 (10), e2304761. 10.1002/advs.202304761 38145353 PMC10933624

[B90] Peruzzotti-Jametti L. Bernstock J. D. Vicario N. Costa A. S. H. Kwok C. K. Leonardi T. (2018). Macrophage-derived extracellular succinate licenses neural stem cells to suppress chronic neuroinflammation. Cell Stem Cell 22 (3), 355–368.e313. 10.1016/j.stem.2018.01.020 29478844 PMC5842147

[B91] Peters A. Rabe P. Liebing A. D. Krumbholz P. Nordström A. Jäger E. (2022). Hydroxycarboxylic acid receptor 3 and GPR84 - two metabolite-sensing G protein-coupled receptors with opposing functions in innate immune cells. Pharmacol. Res. 176, 106047. 10.1016/j.phrs.2021.106047 34968686

[B92] Piras C. Pibiri M. Conte S. Ferranti G. Leoni V. P. Liggi S. (2022). Metabolomics analysis of plasma samples of patients with fibromyalgia and electromagnetic sensitivity using GC-MS technique. Sci. Rep. 12 (1), 21923. 10.1038/s41598-022-25588-2 36535959 PMC9763344

[B93] Qian Z. Xia M. Zhao T. Li Y. Li G. Zhang Y. (2024). ACOD1, rather than itaconate, facilitates p62-mediated activation of Nrf2 in microglia post spinal cord contusion. Clin. Transl. Med. 14 (4), e1661. 10.1002/ctm2.1661 38644791 PMC11033726

[B94] Qin Q. Wang D. Qu Y. Li J. An K. Mao Z. (2025). Enhanced glycolysis-derived lactate promotes microglial activation in parkinson's disease via histone lactylation. NPJ Park. Dis. 11 (1), 3. 10.1038/s41531-024-00858-0 39753581 PMC11698869

[B95] Qiu T. Zhou Y. Hu L. Shan Z. Zhang Y. Fang Y. (2023). 2-Deoxyglucose alleviates migraine-related behaviors by modulating microglial inflammatory factors in experimental model of migraine. Front. Neurol. 14, 1115318. 10.3389/fneur.2023.1115318 37090989 PMC10117646

[B96] Rahimi K. Abbaszadeh M. Bakhtazad S. Ghotbeddin Z. (2024). Effects of dimethyl itaconate on expressions of NGFI-A and NGFI-B and inflammatory cytokines in the spinal cord in the formalin test. Brain Commun. 6 (6), fcae397. 10.1093/braincomms/fcae397 39568551 PMC11577613

[B97] Ramírez-Tejero J. A. Durán-González E. Martínez-Lara A. Lucena Del Amo L. Sepúlveda I. Huancas-Díaz A. (2023). Microbiota and mitochondrial sex-dependent imbalance in fibromyalgia: a pilot descriptive study. Neurol. Int. 15 (3), 868–880. 10.3390/neurolint15030055 37489361 PMC10366818

[B98] Reid P. Scherer K. Halasz D. Simal A. L. Tang J. Zaheer F. (2025). Astrocyte neuronal metabolic coupling in the anterior cingulate cortex of mice with inflammatory pain. Brain Behav. Immun. 125, 212–225. 10.1016/j.bbi.2024.12.025 39694343

[B99] Ren J. Yu L. Lin J. Ma L. Gao D. S. Sun N. (2022). Dimethyl itaconate inhibits neuroinflammation to alleviate chronic pain in mice. Neurochem. Int. 154, 105296. 10.1016/j.neuint.2022.105296 35121012

[B100] Ruan Y. Ling J. Ye F. Cheng N. Wu F. Tang Z. (2021). Paeoniflorin alleviates CFA-induced inflammatory pain by inhibiting TRPV1 and succinate/SUCNR1-HIF-1α/NLPR3 pathway. Int. Immunopharmacol. 101 (Pt B), 108364. 10.1016/j.intimp.2021.108364 34844873

[B101] Sangineto M. Ciarnelli M. Cassano T. Radesco A. Moola A. Bukke V. N. (2023). Metabolic reprogramming in inflammatory microglia indicates a potential way of targeting inflammation in alzheimer's disease. Redox Biol. 66, 102846. 10.1016/j.redox.2023.102846 37586250 PMC10457454

[B102] Saraiva A. L. Veras F. P. Peres R. S. Talbot J. de Lima K. A. Luiz J. P. (2018). Succinate receptor deficiency attenuates arthritis by reducing dendritic cell traffic and expansion of T(h)17 cells in the lymph nodes. Faseb J. 32 (12), 6560–6574. 10.1096/fj.201800285 29894669

[B103] Sergeeva N. S. Krokhicheva P. A. Sviridova I. K. Goldberg M. A. Khayrutdinova D. R. Akhmedova S. A. (2025). Hyaluronan-containing injectable magnesium-calcium phosphate cements demonstrated improved performance, cytocompatibility, and ability to support osteogenic differentiation *in vitro* . Int. J. Mol. Sci. 26 (14), 6624. 10.3390/ijms26146624 40724884 PMC12294418

[B104] Shen J. Wang C. Ying J. Xu T. McAlinden A. O'Keefe R. J. (2019). Inhibition of 4-aminobutyrate aminotransferase protects against injury-induced osteoarthritis in mice. JCI Insight 4 (18), e128568. 10.1172/jci.insight.128568 31534049 PMC6795381

[B105] Shen J. Chen A. Cai Z. Chen Z. Cao R. Liu Z. (2022). Exhausted local lactate accumulation via injectable nanozyme-functionalized hydrogel microsphere for inflammation relief and tissue regeneration. Bioact. Mater. 12, 153–168. 10.1016/j.bioactmat.2021.10.013 35310385 PMC8897073

[B106] Silva Santos Ribeiro P. Willemen H. Versteeg S. Martin Gil C. Eijkelkamp N. (2023). NLRP3 inflammasome activation in sensory neurons promotes chronic inflammatory and osteoarthritis pain. Immunother. Adv. 3 (1), ltad022. 10.1093/immadv/ltad022 38047118 PMC10691442

[B107] Škandík M. Friess L. Vázquez-Cabrera G. Keane L. Grabert K. Cruz De Los Santos M. (2025). Age-associated microglial transcriptome leads to diminished immunogenicity and dysregulation of MCT4 and P2RY12/P2RY13 related functions. Cell Death Discov. 11 (1), 16. 10.1038/s41420-025-02295-1 39828750 PMC11743796

[B108] Sun Q. Hu T. Zhang Y. Wang X. Liu J. Chen W. (2022). IRG1/itaconate increases IL-10 release to alleviate mechanical and thermal hypersensitivity in mice after nerve injury. Front. Immunol. 13, 1012442. 10.3389/fimmu.2022.1012442 36311727 PMC9612919

[B109] Swain A. Bambouskova M. Kim H. Andhey P. S. Duncan D. Auclair K. (2020). Comparative evaluation of itaconate and its derivatives reveals divergent inflammasome and type I interferon regulation in macrophages. Nat. Metab. 2 (7), 594–602. 10.1038/s42255-020-0210-0 32694786 PMC7378276

[B110] Swanson K. V. Deng M. Ting J. P. (2019). The NLRP3 inflammasome: molecular activation and regulation to therapeutics. Nat. Rev. Immunol. 19 (8), 477–489. 10.1038/s41577-019-0165-0 31036962 PMC7807242

[B111] Tang J. Bair M. Descalzi G. (2021). Reactive astrocytes: critical players in the development of chronic pain. Front. Psychiatry 12, 682056. 10.3389/fpsyt.2021.682056 34122194 PMC8192827

[B112] Trauelsen M. Hiron T. K. Lin D. Petersen J. E. Breton B. Husted A. S. (2021). Extracellular succinate hyperpolarizes M2 macrophages through SUCNR1/GPR91-mediated Gq signaling. Cell Rep. 35 (11), 109246. 10.1016/j.celrep.2021.109246 34133934

[B113] Tyagi S. Higerd-Rusli G. P. Ghovanloo M. R. Dib-Hajj F. Zhao P. Liu S. (2024). Compartment-specific regulation of Na(V)1.7 in sensory neurons after acute exposure to TNF-α. Cell Rep. 43 (2), 113685. 10.1016/j.celrep.2024.113685 38261513 PMC10947185

[B114] Ursini F. De Giorgi A. D'Onghia M. De Giorgio R. Fabbian F. Manfredini R. (2021). Chronobiology and chronotherapy in inflammatory joint diseases. Pharmaceutics 13 (11), 1832. 10.3390/pharmaceutics13111832 34834246 PMC8621834

[B115] Wang Y. Guo Y. R. Liu K. Yin Z. Liu R. Xia Y. (2017). KAT2A coupled with the α-KGDH complex acts as a histone H3 succinyltransferase. Nature 552 (7684), 273–277. 10.1038/nature25003 29211711 PMC5841452

[B116] Wang L. Zhang Y. Kiprowska M. Guo Y. Yamamoto K. Li X. (2021). Diethyl succinate modulates microglial polarization and activation by reducing mitochondrial fission and cellular ROS. Metabolites 11 (12), 854. 10.3390/metabo11120854 34940612 PMC8705220

[B117] Wang G. Li Z. Han W. Tian Q. Liu C. Jiang S. (2025). Itaconate promotes mitophagy to inhibit neuronal ferroptosis after subarachnoid hemorrhage. Apoptosis 30 (3-4), 991–1004. 10.1007/s10495-025-02077-1 39924585

[B118] Warfield A. E. Prather J. F. Todd W. D. (2021). Systems and circuits linking chronic pain and circadian rhythms. Front. Neurosci. 15, 705173. 10.3389/fnins.2021.705173 34276301 PMC8284721

[B119] Weibel N. Curcio M. Schreiber A. Arriaga G. Mausy M. Mehdy J. (2024). Engineering a novel probiotic toolkit in *Escherichia coli* nissle 1917 for sensing and mitigating gut inflammatory diseases. ACS Synth. Biol. 13 (8), 2376–2390. 10.1021/acssynbio.4c00036 39115381 PMC11334186

[B120] Wen Z. H. Sung C. S. Lin S. C. Yao Z. K. Lai Y. C. Liu Y. W. (2023). Intra-articular lactate dehydrogenase A inhibitor oxamate reduces experimental osteoarthritis and nociception in rats via possible alteration of glycolysis-related protein expression in cartilage tissue. Int. J. Mol. Sci. 24 (13), 10770. 10.3390/ijms241310770 37445948 PMC10341729

[B121] Wen Z. H. Wu Z. S. Cheng H. J. Huang S. Y. Tang S. H. Teng W. N. (2025). Intrathecal fumagillin alleviates chronic neuropathy-induced nociceptive sensitization and modulates spinal astrocyte-neuronal glycolytic and angiogenic proteins. Mol. Neurobiol. 62 (1), 246–263. 10.1007/s12035-024-04254-w 38837104

[B122] Winter M. Nait Eldjoudi A. Guette C. Hondermarck H. Bourette R. P. Fovez Q. (2023). Mitochondrial adaptation decreases drug sensitivity of persistent triple negative breast cancer cells surviving combinatory and sequential chemotherapy. Neoplasia 46, 100949. 10.1016/j.neo.2023.100949 37956532 PMC10661600

[B123] Wu K. K. (2023). Extracellular succinate: a physiological messenger and a pathological trigger. Int. J. Mol. Sci. 24 (13), 11165. 10.3390/ijms241311165 37446354 PMC10342291

[B124] Xia N. Madore V. Albalakhi A. Lin S. Stimpson T. Xu Y. (2023). Microglia-dependent neuroprotective effects of 4-octyl itaconate against rotenone-and MPP+-induced neurotoxicity in parkinson's disease. Sci. Rep. 13 (1), 15539. 10.1038/s41598-023-42813-8 37730914 PMC10511514

[B125] Xiao M. Yang H. Xu W. Ma S. Lin H. Zhu H. (2012). Inhibition of α-KG-dependent histone and DNA demethylases by fumarate and succinate that are accumulated in mutations of FH and SDH tumor suppressors. Genes Dev. 26 (12), 1326–1338. 10.1101/gad.191056.112 22677546 PMC3387660

[B126] Xiong H. Y. Hendrix J. Schabrun S. Wyns A. Campenhout J. V. Nijs J. (2024). The role of the brain-derived neurotrophic factor in chronic pain: links to central sensitization and neuroinflammation. Biomolecules 14 (1), 71. 10.3390/biom14010071 38254671 PMC10813479

[B127] Xu J. Zheng Y. Zhao Y. Zhang Y. Li H. Zhang A. (2022). Succinate/IL-1β signaling axis promotes the inflammatory progression of endothelial and exacerbates atherosclerosis. Front. Immunol. 13, 817572. 10.3389/fimmu.2022.817572 35273600 PMC8901997

[B128] Yan X. L. Liu X. C. Zhang Y. N. Du T. T. Ai Q. Gao X. (2022). Succinate aggravates intestinal injury in mice with necrotizing enterocolitis. Front. Cell Infect. Microbiol. 12, 1064462. 10.3389/fcimb.2022.1064462 36519131 PMC9742382

[B129] Yang K. Fan M. Wang X. Xu J. Wang Y. Tu F. (2022). Lactate promotes macrophage HMGB1 lactylation, acetylation, and exosomal release in polymicrobial sepsis. Cell Death Differ. 29 (1), 133–146. 10.1038/s41418-021-00841-9 34363018 PMC8738735

[B130] Yang Y. Wang J. Huang S. Li M. Chen J. Pei D. (2024). Bacteria-responsive programmed self-activating antibacterial hydrogel to remodel regeneration microenvironment for infected wound healing. Natl. Sci. Rev. 11 (4), nwae044. 10.1093/nsr/nwae044 38440214 PMC10911815

[B131] Yuan Y. Zhao Y. Shen M. Wang C. Dong B. Xie K. (2022). Spinal NLRP3 inflammasome activation mediates IL-1β release and contributes to remifentanil-induced postoperative hyperalgesia by regulating NMDA receptor NR1 subunit phosphorylation and GLT-1 expression in rats. Mol. Pain 18, 17448069221093016. 10.1177/17448069221093016 35322721 PMC9703502

[B132] Zhang P. Moye L. S. Southey B. R. Dripps I. Sweedler J. V. Pradhan A. (2019). Opioid-induced hyperalgesia is associated with dysregulation of circadian rhythm and adaptive immune pathways in the mouse trigeminal ganglia and nucleus accumbens. Mol. Neurobiol. 56 (12), 7929–7949. 10.1007/s12035-019-01650-5 31129808 PMC6842091

[B133] Zhang J. Si J. Liang R. Lu Y. Shang H. Li X. (2022). Ligand-gated ion channel P2X7 regulates hypoxia-induced factor-1α mediated pain induced by dental pulpitis in the medullary dorsal horn. Front. Mol. Neurosci. 15, 1015751. 10.3389/fnmol.2022.1015751 36385758 PMC9644926

[B134] Zhang X. Lyu D. Li S. Xiao H. Qiu Y. Xu K. (2024). Discovery of a SUCNR1 antagonist for potential treatment of diabetic nephropathy: *in silico* and *in vitro* studies. Int. J. Biol. Macromol. 268 (Pt 2), 131898. 10.1016/j.ijbiomac.2024.131898 38677680

[B135] Zhang Y. Zhang S. Yang L. Zhang Y. Cheng Y. Jia P. (2025). Lactate modulates microglial inflammatory responses through HIF-1α-mediated CCL7 signaling after cerebral ischemia in mice. Int. Immunopharmacol. 146, 113801. 10.1016/j.intimp.2024.113801 39675197

[B136] Zhao K. An R. Xiang Q. Li G. Wang K. Song Y. (2021). Acid-sensing ion channels regulate nucleus pulposus cell inflammation and pyroptosis via the NLRP3 inflammasome in intervertebral disc degeneration. Cell Prolif. 54 (1), e12941. 10.1111/cpr.12941 33111436 PMC7791185

[B137] Zhao B. Zhang Q. He Y. Cao W. Song W. Liang X. (2024). Targeted metabolomics reveals the aberrant energy status in diabetic peripheral neuropathy and the neuroprotective mechanism of traditional Chinese medicine JinMaiTong. J. Pharm. Anal. 14 (2), 225–243. 10.1016/j.jpha.2023.09.007 38464790 PMC10921333

[B138] Zhao N. Yi M. Zhang L. J. Zhang Q. X. Yang L. (2025). 4-Octyl itaconate attenuates neuroinflammation in experimental autoimmune encephalomyelitis Via regulating microglia. Inflammation 48 (1), 151–164. 10.1007/s10753-024-02050-1 38761250

[B139] Zou Y. Bai X. H. Kong L. C. Xu F. F. Ding T. Y. Zhang P. F. (2022). Involvement of histone lysine crotonylation in the regulation of nerve-injury-induced neuropathic pain. Front. Immunol. 13, 885685. 10.3389/fimmu.2022.885685 35911694 PMC9329947

